# GABARAPL1 Inhibits EMT Signaling through SMAD-Tageted Negative Feedback

**DOI:** 10.3390/biology10100956

**Published:** 2021-09-24

**Authors:** Marine Jacquet, Eric Hervouet, Timothée Baudu, Michaël Herfs, Chloé Parratte, Jean-Paul Feugeas, Valérie Perez, Célia Reynders, Marie Ancion, Marc Vigneron, Aurélie Baguet, Michaël Guittaut, Annick Fraichard, Gilles Despouy

**Affiliations:** 1 Université Bourgogne Franche-Comté, INSERM, EFS BFC, UMR1098, Interactions Hôte-Greffon-Tumeur/Ingénierie Cellulaire et Génique, F-25000 Besançon, France; marine.jacquet02@edu.univ-fcomte.fr (M.J.); eric.hervouet@univ-fcomte.fr (E.H.); timothee.baudu@edu.univ-fcomte.fr (T.B.); chloe.parratte@edu.univ-fcomte.fr (C.P.); jean-paul.feugeas@univ-fcomte.fr (J.-P.F.); valerie.perez@univ-fcomte.fr (V.P.); aurelie.baguet@univ-fcomte.fr (A.B.); michael.guittaut@univ-fcomte.fr (M.G.); annick.fraichard@univ-fcomte.fr (A.F.); 2 DImaCellplatform, Université Bourgogne Franche-Comté, F-25000 Besançon, France; 3 EPIGENExp, Université Bourgogne Franche-Comté, F-25000 Besançon, France; 4 Laboratory of Experimental Pathology, GIGA-Cancer, University of Liege, 4000 Liege, Belgium; m.herfs@ulg.ac.be (M.H.); celia.reynders@uliege.be (C.R.); marie.ancion@uliege.be (M.A.); 5 Team Replisome Dynamics and Cancer, UMR7242 Biotechnologie et Signalisation Cellulaire, Ecole Supérieure de Biotechnologie de Strasbourg, CNRS-Université de Strasbourg, F-67412 Illkirch, France; marc.vigneron@unistra.fr

**Keywords:** autophagy, ATG8, GABARAPL1, cancer, EMT, SMAD

## Abstract

**Simple Summary:**

Epithelial–mesenchymal transition (EMT) is involved in metastasis formation, chemoresistance, apoptosis resistance, and acquisition of stem cell properties, making this process an attractive target in cancer. However, direct targeting of EMT remains challenging. Autophagy—an intracellular mechanism—has been noted to be involved in the regulation of EMT—mainly by its involvement in the degradation of EMT actors, explaining why understanding of how autophagy could regulate EMT might be promising in the development of new cancer therapies. Here, we found that GABARAPL1—an autophagy-related gene—was increased in human NSCLC mesenchymal tumors compared to epithelial tumors, and induction of EMT in an A549 lung cancer cell line by TGF-β/TNF-α cytokines also led to an increase in GABARAPL1 expression. This regulation could involve the EMT-related transcription factors of the SMAD family. To understand the role of GABARAPL1 in EMT regulation in lung cancer cells, A549 KO GABARAPL1 were designed and used to investigate whether GABARAPL1 could inhibit EMT via its involvement in SMAD degradation. The results indicate that GABARAPL1-mediated autophagic degradation could intervene as a negative EMT-regulatory loop.

**Abstract:**

The pathway of selective autophagy, leading to a targeted elimination of specific intracellular components, is mediated by the ATG8 proteins, and has been previously suggested to be involved in the regulation of the Epithelial–mesenchymal transition (EMT) during cancer’s etiology. However, the molecular factors and steps of selective autophagy occurring during EMT remain unclear. We therefore analyzed a cohort of lung adenocarcinoma tumors using transcriptome analysis and immunohistochemistry, and found that the expression of *ATG8* genes is correlated with that of EMT-related genes, and that GABARAPL1 protein levels are increased in EMT+ tumors compared to EMT- ones. Similarly, the induction of EMT in the A549 lung adenocarcinoma cell line using TGF-β/TNF-α led to a high increase in GABARAPL1 expression mediated by the EMT-related transcription factors of the SMAD family, whereas the other ATG8 genes were less modified. To determine the role of GABARAPL1 during EMT, we used the CRISPR/Cas9 technology in A549 and ACHN kidney adenocarcinoma cell lines to deplete GABARAPL1. We then observed that GABARAPL1 knockout induced EMT linked to a defect of GABARAPL1-mediated degradation of the SMAD proteins. These findings suggest that, during EMT, GABARAPL1 might intervene in an EMT-regulatory loop. Indeed, induction of EMT led to an increase in GABARAPL1 levels through the activation of the SMAD signaling pathway, and then GABARAPL1 induced the autophagy-selective degradation of SMAD proteins, leading to EMT inhibition.

## 1. Introduction

Epithelial–mesenchymal transition (EMT) has been shown to play a major role during cancer cell metastasis, the escape of the immune system by cancer cells, and the acquisition of apoptosis resistance and cellular stemness properties, leading to anticancer drug resistance [1]. These data explain why the choice to target EMT has emerged as a new anticancer therapeutic strategy during the past decade. EMT is a plastic and reversible biological process induced by cellular stresses and growth factors found in the tumor microenvironment, such as TGF-β, which is the most described growth factor involved in EMT induction. TGF-β activates the SMAD-dependent and SMAD-independent signaling pathways, triggering the activation of EMT transcription factors (EMT-TFs) such as SNAI1, TWIST, and ZEB. EMT-TFs then downregulate key epithelial genes (e.g., E-cadherin/CDH1) and upregulate key mesenchymal genes (e.g., N-cadherin/CDH2, vimentin) [2], leading to the acquisition of mesenchymal cell properties, such as migratory and invasive abilities, a spindle-shaped morphology, and self-renewal capacity [3]. The SMAD-dependent pathway consists of the phosphorylation of SMAD2 and SMAD3 by the TGF-β receptor, and the trimerization of SMAD2, SMAD3, and SMAD4. Then, the protein complex is translocated to the nucleus, where it acts as a transcription factor, upregulating EMT-related genes [4].

Macroautophagy (hereafter referred to as autophagy) is a catabolic pathway leading to the sequestration of misfolded or damaged proteins and/or organelles into autophagosomes, and their subsequent targeting, degradation, and recycling [5,6]. Autophagy was originally described as a non-selective degradation pathway, but different types of selective autophagy have since been discovered, such as autophagy of ubiquitinylated proteins, mitophagy, aggrephagy, etc., [7,8,9]. Selective autophagy involves the family of ATG8 proteins (autophagy-related 8), composed of two subfamilies: the GABARAP subfamily (GABA type A receptor-associated protein), comprising 3 members (GABARAP, GABARAPL1 (GABARAP-like 1), and GABARAPL2 (GABARAP-like 2)); and the MAP-LC3 subfamily (microtubule-associated protein light chain 3), commonly called LC3, including 4 members (LC3A, LC3B, LC3B2, and LC3C) [10,11,12,13,14,15,16,17]. During selective autophagy, ATG8 proteins are recruited and conjugated to a phospholipid on the autophagosome membrane, and can then function as anchor points to specifically recruit the selected proteins and/or organelles to degrade. This recruitment requires an autophagic adaptor containing the conserved AIM sequence (ATG8-interacting motif) found in these proteins [18,19]. These ATG8 proteins are also involved in non-selective autophagy (macroautophagy) through the regulation of key steps of the autophagic pathway, such as initiation, elongation, autophagosome transport, and autophagosome/lysosome fusion [20].

Autophagy has been described as presenting a double-edged sword in cancer. Firstly, antitumor autophagy maintains cellular homeostasis and genome stability via the degradation of reactive oxygen species, leading to the inhibition of cellular transformation [21]. During cancer progression, autophagy can indeed exert an antitumor effect via the degradation of oncoproteins, such as BCR-ABL in leukemia or RhoA in lung carcinoma [22,23]. Moreover, autophagy can lead to cancer cell death, necrosis, or senescence [24,25,26], thereby inhibiting cancer cell aggressiveness. However, autophagy can also exert a pro-tumor function, since it can provide nutrients to cancer cells and inhibit stress-induced cell death linked to hypoxia [27]. Autophagy can also protect cancer cells from apoptosis [28], and allows for the maintenance of stem cells’ properties [29], leading to increased cancer cell aggressiveness. Regarding EMT, autophagy also presents a double-edged sword, and the role of autophagy during this process remains unclear. On the one hand, EMT requires autophagy to sustain the viability of the metastatic cells but, on the other hand, autophagy may prevent EMT through the degradation of EMT factors [30,31,32,33,34,35]. 

In this work, we were interested in characterizing the function of ATG8 proteins during EMT, and we found that ATG8 mRNA and proteins were correlated with EMT markers in human lung adenocarcinoma tumors. Moreover, amongst the ATG8 family, GABARAPL1 expression presented the greatest increase in expression during TGF-β/TNF-α-induced EMT in A549 lung adenocarcinoma cells. Finally, we found that GABARAPL1 is involved in the inhibition of EMT via its involvement in the selective degradation of the EMT-related transcription factors of the SMAD family.

## 2. Materials and Methods

### 2.1. Transcriptome Analysis

We used R software (version 3.6.0) (http://www.R-project.org, June 2019) to collect datasets and to carry out all statistical analyses. Computations were performed using the supercomputer facilities of the “Mésocentre de calcul de Franche-Comté” (Besançon, France). A total of 594 transcriptomes produced by RNA-Seq were collected from the Cancer Genome Atlas (TCGA) website (https://portal.gdc.cancer.gov, June 2019) using the TCGAbiolinks R package. Downloaded HT-Seq count files were normalized using the “Deseq-2” R package, using the function “varianceStabilizingTransformation”. For subsequent analyses, we retained 372 samples diagnosed as NOS adenocarcinoma. Correlations between ATG8 and EMT-related genes were globally analyzed via principal component analysis using the FactoMineR package. Correlation circles were drawn using this package. Pearson’s correlation coefficients (r) were calculated to quantify two-by-two correlations. When necessary, RNA levels were sorted into negative and positive populations using the Fisher algorithm. Comparisons between groups were carried out within the general linear regression model (lm function in R).

### 2.2. Reagents and Antibodies

Ammonium chloride (NH_4_Cl, A0171, Sigma-Aldrich, St. Louis, MI, USA), Earl’s balanced salt solution (EBSS, E3024, Sigma-Aldrich), TGFβ (100-21, PeproTech, Rocky Hill, NJ, USA), TNF-α (300-01A, PeproTech), and SIS3HCl (521984-48-5, CliniSciences, Nanterre, France) were used. For the Western blotting experiments, the following antibodies were used: GABARAPL1 (26632S, Cell Signaling, Saint-Cyr-L’École, France), LC3 (LB8918, Sigma, Saint-Quentin-Fallavier, France), N-cadherin (D4R1H, Cell Signaling), E-cadherin (610181, BD Biosciences, Le Pont de Claix, France), SNAI1 (C15D3, Cell Signaling), SMAD2 (5339T, Cell Signaling), SMAD3 (9523T, Cell Signaling), SMAD4 (38454T, Cell Signaling), β-actin (A5060, Sigma-Aldrich), anti-rabbit (BI2413C, P.A.R.I.S, Diagenode), and anti-mouse (BI2407, P.A.R.I.S). For IHC experiments, the following antibodies were used: vimentin (#7902917, Ventana Medical Systems, Illkirch-Graffenstaden, France), GABARAP (SAB1302861, Sigma-Aldrich), and GABARAPL1 (11010-1-AP, ProteinTech, Manchester, UK). For confocal microscope experiments, actin was labeled using phalloidin (P1951, Sigma-Aldrich), and the following antibodies were used: SMAD2 (5339T, Cell Signaling), SMAD2/3 (sc-133098, Santa Cruz Biotechnology, Heidelberg, Germany), and Alexa Fluor 488 goat anti-rabbit (Life Technologies, Rockville, MD, USA, A-11008, 1:500).

### 2.3. Tissue Samples and Immunohistochemistry (IHC) 

Paraffin-embedded tissue specimens of NSCLCs were collected in collaboration with the Tissue Biobank of the University of Liege (Liege, Belgium). The protocol was approved by the Ethics Committee of the University Hospital of Liege. The initial diagnosis of each case was confirmed by experienced histopathologists. IHC analysis was performed using a standard protocol previously described in [36]. All samples were first classified into two groups: EMT+ and EMT−, based on the presence or absence of anti-vimentin immunoreactivity displayed by tumor cells. Regarding the other proteins analyzed by IHC, as previously described [37], the intensity and the extent of the staining was assessed using an arbitrary scale ranging from 0 to 3. The 2 scores were then multiplied to obtain a global score between 0 and 9. All immune-labeled tissues were evaluated by two experienced histopathologists.

### 2.4. Cell Culture and Treatments

A549 (lung adenocarcinoma, ATCC CCL-185) and ACHN (kidney adenocarcinoma, ATCC CRL-1611) cancer cells were cultured in DMEM 1g/l glucose (L0066, Dominique Dutscher, Bernolsheim, France) supplemented with 10% FBS (702774C, Corning, Amsterdam, The Netherlands) and 100 µg/mL penicillin/streptomycin (PAA, P11-010) in a 5% CO_2_ incubator at 37 °C. To inhibit autophagy, cells were treated with 50 mM NH_4_Cl for 2 h. To induce EMT, cells were co-treated with 5 ng/mL TGF-β and 20 ng/mL TNF-α from 10 to 24 h. 

### 2.5. Generation of KO Cell Lines Using the CRISPR/Cas9 Gene Editing Technology

A549 KO GABARAPL1 cell lines were generated using the CRISPR/Cas9 gene editing technology. A549 wild-type cells were transfected with the pCAS9-GFP-sgRNA GABARAPL1a (sgRNA sequence: CACCGCCGGAAGAAATATCCGGAC) or the pCAS9-GFP-sgRNA GABARAPL1b (sgRNA sequence: AAACGTCCGGATATTTCTTCCGGC), targeting two distinct sites of the first exon of GABARAPL1, leading to the generation of the A549 KO GABARAPL1 c1 (A549 KO GL1 c1) and A549 KO GABARAPL1 c3 (A549 KO GL1 c3) cell lines, respectively. ACHN wild-type cells were transfected with the pCAS9-GFP-sgRNA GABARAPL1b plasmid, leading to the generation of the ACHN KO GABARAPL1 cA and ACHN KO GABARAPL1 cB cell lines. A549c and ACHNc cells were generated following transfection with the pCAS9-GFP-sgRNA control plasmid. The day after transfection, GFP-positive cells were sorted by FACS (SH800, SONY) and colonies were expanded before being screened for the loss of GABARAPL1 expression by Western blotting and sequencing. For A549c and ACHNc control cells, all of the GFP-positive cells were pooled and, therefore, these cell lines correspond to polyclonal cell lines.

For transient transfection, the pCAS9-GFP-sgRNA control or the pCAS9-GFP-sgRNA GABARAPL1 plasmids were transfected using the jetPRIME reagent (Polyplus-transfection, 114-07), according to the manufacturer’s protocol. 

For sequencing analysis, genomic DNA from A549c, ACHNc, and KO GL1 clones was extracted (DNeasy blood and tissue kit, 69504) and amplified by PCR (for A549 KO GL1 c1: F- CGGTGCATCATGAAGTTCCA and R- CTCCGCTCCTCTACACTCAC; and for A549 KO GL1 c3, and ACHN KO GL1 cA and cB: F- GCCCTGCGGTGCATCAT and R- CATCCCCTCCGCTCCTCTACACT). PCR products were cloned into the p.JET1.2 vector (Thermo Fisher, 10659920) following the manufacturer’s instructions. JM109 bacteria were transformed with the ligation product and grown on ampicillin LB medium. At least 10 colonies were sequenced (SANGER sequencing on 3130 GA Applied Biosystems).

### 2.6. Cells Transfection

A549 cells were plated in 6-well plates at a density of 200,000 cells/well. For transient transfection, the pCDNA3.1_control or the pCDNA3.1_Flag-GABARAPL1-6His plasmids [1] were transfected using the jetPRIME reagent (114-07, Polyplus-transfection), according to the manufacturer’s protocol. 

### 2.7. Cell Proliferation 

A549 cells were plated in 96-well plates at a density of 1500 cells/well. Cell proliferation experiments were then conducted for 5 days using MTT (3-(4,5-dimethylthiazol-2-yl)-2,5-diphenyl tetrazolium bromide) (Sigma-Aldrich, M2128). Each day, after removing the cell supernatant, 100 µL of a 100 mM MTT solution diluted in PBS was added to the cells. After a 2 h incubation at 37 °C and 5% CO_2_, formazan crystals were dissolved in 50 µL of DMSO (dimethyl sulfoxide) (Euromedex, UD8050-A), and the absorbance was quantified at 549 nm using microplate reader (Multiskan FC, Thermo Scientific, Asnières sur Seine, France).

### 2.8. Cell Invasion Assays

Cell invasion was evaluated using modified Boyden chambers (ThinCert for 24-well plates, 8 μm pore size, Greiner Bio-one, 438122). Boyden chambers were coated with 50 µL of ECM (extra cellular matrix) gel from Engelbreth-Holm-Swarm murine sarcoma (Sigma-Aldrich, E1270, 1 mg/mL) diluted in DMEM for 5 h at 37 °C. A total of 100,000 cells in 150 µL of serum-free DMEM were seeded into the upper chamber, and 500 µL of complete culture medium was added to the lower compartment. After a 24 h incubation in a 5% CO_2_ atmosphere at 37 °C, cells present in the upper compartment were removed. Then, cells in the lower compartment and cells that had streamed across the membrane were rinsed with PBS, fixed for 5 min with absolute ethanol, and then stained with crystal violet (0.5% in 2% ethanol) for 10 min. Filters and wells were then washed with distilled water, and cell density was counted using the EVOS XL Core microscope.

### 2.9. Cell Migration Assays

Cells migration was evaluated using the scratch wound healing assay, wherein 50,000 cells were plated in 96-well plates. The next day, the scratch was made using the IncuCyte^®^ WoundMaker. The cells were then photographed every two hours for one day, and wound healing was quantified using the percentage of the confluence of the wound by the IncuCyte every 2 h for 24 h. 

Cell migration was also evaluated using modified Boyden chambers (ThinCert for 24-well plates, 8 μm pore size, Greiner Bio-one, 438122). A total of 100,000 cells in 150 µL of serum-free DMEM were seeded into the upper chamber, and 500 µL of complete culture medium was added to the lower compartment. After a 24 h incubation in a 5% CO_2_ atmosphere at 37 °C, cells present in the upper compartment were removed. Then, cells in the lower compartment and cells that had streamed through the membrane were rinsed with PBS, fixed for 5 min with absolute ethanol, and then stained with crystal violet (0.5% in 2% ethanol) for 10 min. Filters and wells were then washed with distilled water, and cell density was counted using the EVOS XL Core microscope.

### 2.10. Western Blotting

Cells were washed by adding cold PBS, and then scraped and lysed in SB 1X (45 mM Tris-HCl, pH 7.6, 10% Glycerol, 2% SDS, 1.5% 2-mercaptoethanol, 0.001% bromophenol blue) or RIPA (50 mM Tris-HCl, pH 8, 150 mM NaCl, 1% Triton X100, 0.5% DOCA, 0.1% SDS) buffer supplemented with 1X protease inhibitors (P8340, Sigma-Aldrich). Protein lysates were sonicated for 5 s and boiled to 95 °C for 5 min before being loaded on a 4–12% TGX Gel (TGX FastCast Acrylamide Solution, Bio-Rad, Marnes-la-Coquette, France) according to the manufacturer’s instructions. Proteins were transferred onto a polyvinylidene difluoride (PVDF) membrane (162-0177, Bio-Rad) using the Trans-Blot Turbo System (Bio-Rad). The membrane was blocked with 5% nonfat milk or BSA (bovine serum albumin) in Tris-buffered saline with Tween 20 (20 mM Tris-HCl, pH 7.6, 137 mM NaCl 0.1% Tween 20) and then incubated with primary antibodies overnight at 4 °C. Membranes were washed 3 times with TBS-Tween 20 0.1%, and then incubated with secondary anti-rabbit or anti-mouse HRP-conjugated antibody according to the manufacturer’s instructions (BI2407, BI2413C, Paris, France). The membrane was then washed 3 times with TBS-Tween 20 0.1% and incubated with Clarity Western Cl substrate (1705051, Bio-Rad), and chemiluminescence was monitored using a Bio-Rad ChemiDoc XRS+.

### 2.11. Quantitative RT-PCR

Total RNAs were isolated from cells using the Tri Reagent (Molecular Research Center, TR-118) in accordance with the manufacturer’s instructions. Reverse transcription was performed using M-MLV (M-1302, Sigma-Aldrich) reverse transcriptase and 2 µg of total RNA. Quantitative PCR (qPCR) was performed in triplicate using a StepOne Real-Time PCR System (Applied Biosystems) and the SYBR qPCR Premix Ex Taq (Tli RNase H Plu (TAKRR420W, Takara), according to the manufacturer’s instructions. The following specific primers were used: GABARAP (F-GCCTTTCCCATCCTGCTGTA and R- GGAAGGGATTGCTGGGTTCT), GABARAPL1 (F-CCCTCCCTTGGTTATCATCCA and R-ACTCCCACCCCACAAAATCC), GABARAPL2 (F-TAGTGGCCACAATGACCAGA and R-TGAACACAGCTTTGGTCCAG), LC3B (F-CGGAAAGCAGCAGTGTACCA and R-GGCAGAAGGGAGTGTGTCTGA), SNAI1 (F- CGCGCTCTTTCCTCGTCAG and R- TCCCAGATGAGCATTGGCAG), E-cadherin (F-AGGATGGTGTAAGCGATGGC and R-CGGGAATGCAGTTGAGGATC), N-cadherin (F-CTCATATGGTGGAGCTGTGGC and R-TGTGGGAATCCGACGAATG), SMAD2 (F- ACCGAAATGCCACGGTAGAA and R- TGGGGCTCTGCACAAAGAT), SMAD3 (F- GCCTGTGCTGGAACATCATC and R- TTGCCCTCATGTGTGCTCTT), and SMAD4 (F- CATCCTGCTCCTGAGTATTGG and R- GGGTCCACGTATCCATCAAC). H3-3A (histone 3-3A) was used as housekeeping gene (F-GCTAGCTGGATGTCTTTTGG and R-GTGGTAAAGCACCCAGGAA). 

### 2.12. Confocal Microscopy

A549 cells were plated on coverslips in 24-well plates at a density of 20,000 cells/well. For transient transfection, the ptf-LC3 (137624, Addgene) plasmid was transfected using the jetPRIME reagent (114-07, Polyplus-transfection) according to the manufacturer’s protocol. The next day, the cells were washed with cold PBS and fixed with 4% PFA (paraformaldehyde) in PBS for 15 min at room temperature, and then mounted using Fluoromount Aqueous Mounting Medium (F4680, Sigma-Aldrich). Images were acquired using a Zeiss LSM 800 Airyscan confocal microscope (63× objective). Red and yellow puncta were counted using the “green and red puncta colocalization tool” designed for Image J. For each experiment, 20 cells were randomly selected. 

For phalloidin staining, cells were treated, or not, with TGF-β/TNF-α for 24 h. The cells were washed with cold PBS, fixed using 4% PFA in PBS for 15 min, and stained with 10 µM phalloidin for 10 min. The cells were washed 3 times, their nuclei were stained using DAPI (4′,6′-diamidino-2-phénylindole), and cells were then mounted using Fluoromount Aqueous Mounting Medium. The cells were analyzed using a Zeiss LSM 800 Airyscan confocal microscope. For each experiment, 10 cells were randomly selected, and the area of the cells was measured using Image J software. 

For the immunofluorescence experiment, A549c cells were plated on coverslips in 24-well plates at a density of 10,000 cells/well. For transient transfection, the pGFP-LC3 plasmid, kindly provided by Dr. Elazar (Weizmann Institute, Rehovot Israël), was transfected using the jetPRIME reagent, according to the manufacturer’s protocol. The next day, the different treatments were added. Then, the cells were washed with cold PBS, fixed with 4% PFA in PBS for 15 min at room temperature and, permeabilized using PBS 0.1% triton-X100. The saturation was performed using 5% BSA for 1 h at 37 °C, and the primary antibody was diluted in 1% BSA and incubated overnight at 4 °C. The secondary antibody—an Alexa Fluor 488 goat anti-rabbit—was incubated for 1 h at 37 °C, and the nuclei were stained using DAPI, before the cells were mounted as previously described. The cells were then analyzed using a Zeiss LSM 800 Airyscan confocal microscope. For each experiment, 15 cells were randomly selected, and the colocalization was analyzed using the “JACoP” plugin and the “colocalization based on distance between center of mass” method designed for Image J. 

For the in situ proximity ligation assay (P-LISA), A549c cells were plated on coverslips in 24-well plates at a density of 10,000 cells/well and permeabilized as previously described. P-LISA staining was performed according to the recommendations of Olink Bioscience using the Duolink^®^ In Situ Detection Reagents Red Kit (DUO92008, Sigma-Aldrich), as previously described [38]. The nuclei were stained using DAPI and the cells were mounted as previously described. The number of red dots was then counted using Image J software.

### 2.13. GST-Pull down Assay

All GST-tagged proteins were expressed in *Escherichia coli BL21* (DE3) and purified on glutathione–Sepharose beads (Sigma-Aldrich, Cytiva, 17-0756-01) according to the manufacturer’s instructions. For affinity purification of protein interactions, GST and GST-GABARAPL1, bound to glutathione Sepharose beads, were incubated with A549c cell lysates in Tris HCl 20 mM pH 8, NaCl 150 mM, NP40 1%, EDTA 2mM, and PIC 1X lysis buffer for one night at 4 °C under agitation. Bound proteins were then isolated via centrifugation of Sepharose beads at 10,000 g for 1 min, washed 3 times in PBS NaCl 300 mM, eluted by boiling in SB 1X (45 mM Tris- HCl, pH 7.6, 10% Glycerol, 2% SDS, 1.5% 2-mercaptoethanol, 0.001% bromophenol blue), and then subjected to SDS-PAGE separation. Total proteins were detected by stain-free or by immunoblotting using an anti-SMAD2/3 antibody (cell signaling). 

### 2.14. Statistical Analysis

Statistical analyses were carried out using GraphPad Prism statistical software. For all experiments, results are reported as the mean ± S.E.M. of at least three independent experiments. *p*-values *p* * < 0.05, *p* ** < 0.01, and *p* *** < 0.0001 were considered as a threshold for significance, and were calculated using Student’s *t*-test. When the bar is absent, the value is compared to the untreated control cells.

## 3. Results

### 3.1. GABARAPL1 Expression Was Correlated with EMT Markers 

**Figure 1:**
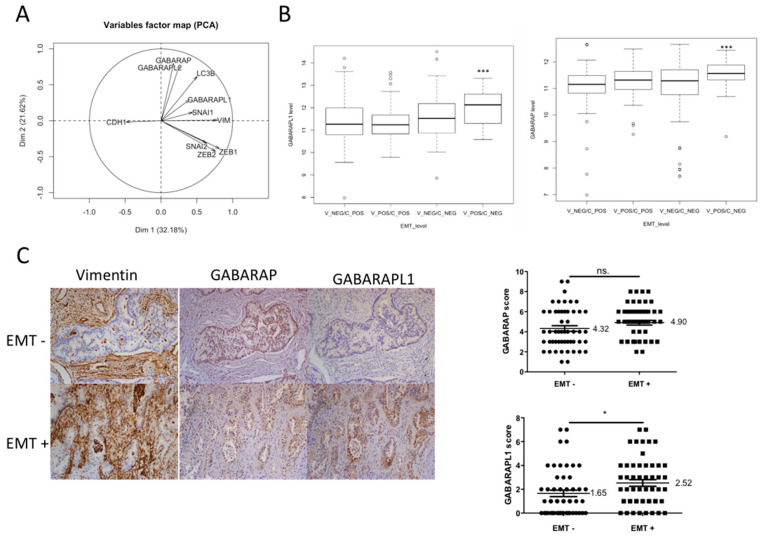
GABARAPL1 correlates with EMT markers in lung adenocarcinomas. (**A**) Correlations between ATG8 and EMT-related gene expression in primary lung adenocarcinoma. Correlation circles were built with the two first components (Dim1 and Dim2) of the principal component analysis (PCA). Dim1 and Dim2 explain 32.18% and 21.62 of variability, respectively. PCA was performed with the RNA-Seq data downloaded from the TCGA data portal (372 samples). (**B**) Boxplots of GABARAPL1 and GABARAP RNA levels according to cadherin 1 (C) and vimentin (V) in the lung carcinoma TCGA database. C and V RNA values were discretized in negative (NEG) and positive (POS), allowing determination of four groups of tumors with increased EMT, from V_NEG/C_POS to V_POS/C_NEG tumors. Indicated *p*-values were obtained after analysis of variance of GABARAPL1 and GABARAP RNA levels in the four groups. (**C**) Immunohistochemical analysis of GABARAP and GABARAPL1 expression in lung adenocarcinoma tumors (60 tumors), divided according to the high vimentin immunoreactivity displayed (EMT+, n = 30), or not (EMT-, n = 30), by tumor cells.

To investigate whether the ATG8 genes might be involved in EMT in lung adenocarcinoma tumors, 372 transcriptomes of lung adenocarcinomas were collected from the TCGA database, and correlations between ATG8 genes and EMT-related genes were studied using principal component analysis. The correlation circles showed that expression of ATG8 genes (*GABARAP*, *GABARAPL1*, *GABARAPL2*, *LC3B*) was positively associated with the expression of EMT-related genes, and negatively correlated with the expression of the epithelial marker CDH1. However, *GABARAPL1* and *LC3B* were the only *ATG8* genes positively associated with both the mesenchymal marker vimentin and the EMT-related transcription factors (SNAI1, SNAI2, ZEB1, ZEB2), but were also negatively correlated with the epithelial marker CDH1. GABARAPL1 provided the most significant correlation with EMT-TFs and mesenchymal markers (Figure 1A). We also compared GABARAP and GABARAPL1 mRNA levels between CDH1-/VIM+ tumors and CDH1+/VIM- tumors, using data obtained from the TCGA database (Figure 1B). We found that GAPARAPL1 was significantly more expressed in CDH1-/VIM+ tumors compared to the CDH1+/VIM- tumors, while other groups (CDH1-/VIM- and CDH1+/VIM+) presented intermediate levels (fold change = 1.5, *p*-value < 10^−6^); this was also true for GABARAP, albeit to a lesser extent (fold change = 1.4, *p*-value < 10^−5^). Next, we investigated the protein levels of GABARAP and GABARAPL1 by IHC in 60 samples of lung adenocarcinomas. First, tumors were classified as EMT- (n = 30) or EMT+ (n = 30) based on their anti-vimentin staining. Then, the expression of GABARAP and GABARAPL1 was analyzed in both groups. As already observed for transcriptome data analysis, no significant difference in terms of GABARAP staining was observed between EMT- and EMT+ tumors (Figure 1C). On the other hand, EMT+ tumors displayed a more intense GABARAPL1 staining than their EMT- counterparts (Figure 1C). Altogether, these interesting results support our hypothesis that GABARAPL1 expression is correlated with classical EMT markers at both the mRNA and protein levels in human lung adenocarcinoma.

### 3.2. TGF-β/TNF-α-Induced EMT Was Correlated with Increased GABARAPL1 Expression

**Figure 2:**
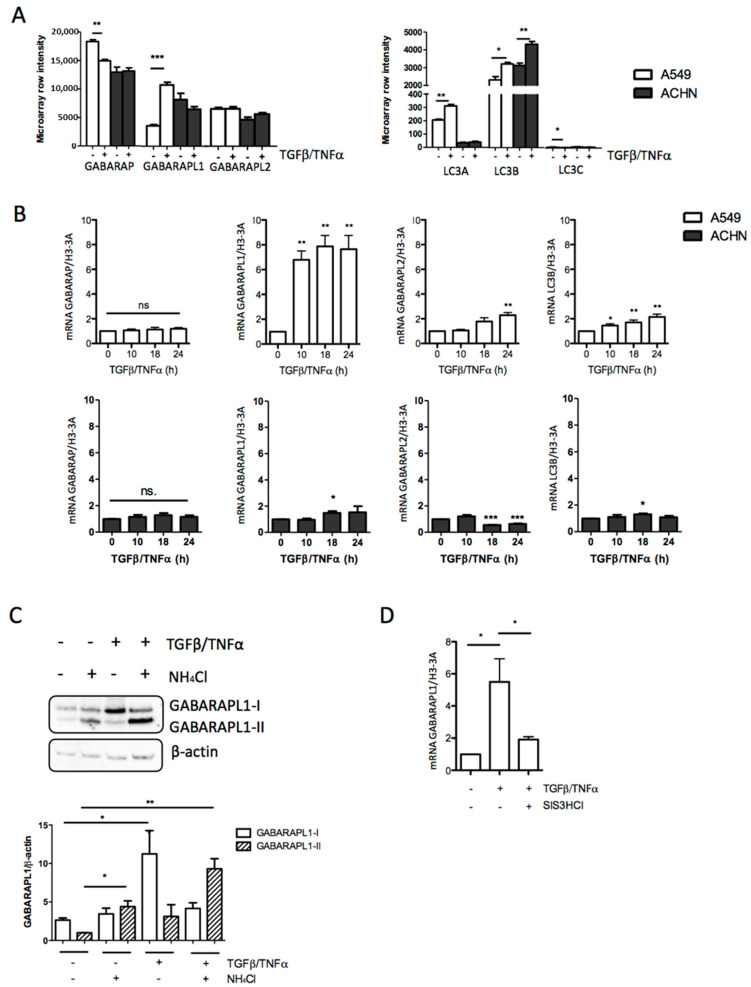
GABARAPL1 expression is increased during TGF-β/TNF-α-induced EMT. (**A**) Transcriptome analysis of ATG8 gene expression. ATG8 gene expression was quantified by microarray in A549 and ACHN cells untreated or treated with TGF-β (5 ng/mL) and TNF-α (20 ng/mL) for 5 days [26]. (**B**) mRNA levels of GABARAP, GABARAPL1, GABARAPL2, and LC3B were measured by qRT-PCR over the course of 0, 10, 18, and 24 h of TGF-β (5 ng/mL)/TNF-α (20 ng/mL)-induced EMT in A549 cells. The values calculated by the ∆∆CT method are relative to the levels of H3-3A, and are expressed as fold change. (**C**) Western blot analysis of GABARAPL1 levels in A549c cells untreated or treated with 50 mM NH_4_Cl for 2 h in the presence or abseΔΔnce of TGF-β 5 ng/mL and TNF-α 20 ng/mL for 24 h. β-actin was used as a loading control. Protein levels were quantified using Image Lab. (**D**) mRNA levels of GABARAPL1 were measured by qRT-PCR in A549c cells. Cells were pretreated or not by the inhibitor of SMAD3 phosphorylation, SIS3HCl (10 μM), for 6 h before induction of EMT by TGF-β (5 ng/mL)/TNF-α (20 ng/mL) for 24 h. The values calculated by the ∆∆CT method are relative to the levels of H3-3A, and are expressed as fold change.

In a previous study from our laboratory [39] EMT was induced in A549 lung adenocarcinoma cells, ACHN kidney adenocarcinoma cells derived from metastatic pleural sites, and MCF10A immortalized breast cells using a combination of TGF-β and TNF-α, and gene expression was analyzed using microarray protocols. TGF-β is a growth factor currently used to induce EMT, while TNF-α is a compound described as enhancing the effect of TGF-β. The analysis of these microarray data showed that, after 5 days of TGF-β/TNF-α-induced EMT, the cells presented a strong mesenchymal phenotype, and it was observed that TGF-β/TNF-α-induced EMT led to a high increase in GABARAPL1 levels (approximately threefold), whereas only slight differences were detected for the other ATG8 genes in A549 cells (Figure 2A). Similarly, in MCF10A cells, the induction of EMT also led to a high increase in GABARAPL1 expression. However, in these cells, the expression of the other ATG8 genes was also increased during EMT, albeit to a lesser extent (Figure S1A). Surprisingly, in ACHN cells, only a weakly significant increase in LC3B levels was observed (Figure 2A). We then wanted to analyze the differences in expression during the first 24 h of EMT induction. Quantitative RT-qPCR experiments showed that GABARAPL1 was highly upregulated between 10 and 24 h of TGF-β/TNF-α treatment in A549 cells, while only a weak increase in GABARAPL2 and LC3B expression was observed (Figure 2B). The increase in GABARAPL1 expression in A549 cells was then confirmed at the protein level after 24 h of treatment with TGF-β/TNF-α in the presence or absence of NH_4_Cl, in order to quantify the total level of GABARAPL1 (the soluble form GABARAPL1-I and the membrane-bound form GABARAPL1-II) (Figure 2C). On the other hand, in ACHN cells, the TGF-β/TNFα treatment only induced a slight increase in GABARAPL1 and LC3B expression at 18 h, and a slight decrease in GABARAPL2 expression (Figure 2B). These results suggest that, during TGF-β/TNF-α-induced EMT, GABARAPL1 is the most regulated gene amongst the ATG8 gene family during EMT, and the increase in GABARAPL1 levels seems to be an early response to TGF-β/TNF-α treatment. We then wanted to determine whether the TGF-β-related signaling pathway regulated GABARAPL1 expression. As previously described, the SMAD signaling pathway has already been shown to be involved in the regulation of ATG5, ATG7, and BECN1 autophagy-related gene expression [28]. To address this question, we inhibited the SMAD signaling pathway using SIS3HCl—an inhibitor of the phosphorylation of SMAD3. We found that the increase in GABARAPL1 expression induced by TGF-β/TNF-α was inhibited in the presence of SIS3HCl at the mRNA and protein levels, suggesting the involvement of the SMAD signaling pathway in the regulation of GABARAPL1 expression (Figure 2D and Figure S1B,C).

### 3.3. Design and Characterization of GABARAPL1 Knockout Cell Lines

**Figure 3:**
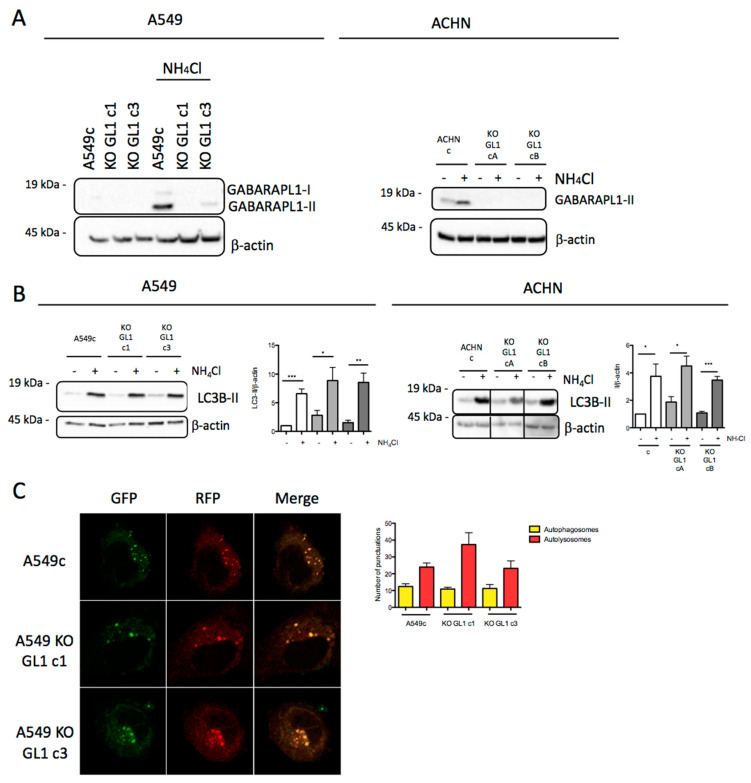
The GABARAPL1 knockout does not modify autophagic flux. (**A**) Western blot analysis of GABARAPL1 levels in A549c, ACHNc, and KO GABARAPL1 cells untreated or treated with 50 mM NH_4_Cl for 2 h. (**B**) Western blotting analysis of LC3B levels in A549c, ACHNc, and KO GABARAPL1 cells cultured in complete medium and treated, or not, with 50 mM NH_4_Cl for 2 h. β-actin was used as a loading control. Protein levels were quantified using Image Lab. (**C**) GFP-RFP-LC3 puncta analysis in A549c and A549 KO GL1 cells transfected with the ptf-LC3 vector and cultured in complete medium, by confocal microscopy. Each picture is representative of a typical staining observed in 20 fields chosen at random. Red and yellow puncta were counted using ImageJ software (green and red puncta colocalization tool). In each group, 20 cells were randomly selected. Scale bar: 5 m.

To characterize the role of GABARAPL1 in EMT signaling, we designed A549 KO GABARAPL1 and ACHN KO GABARAPL1 (KO GL1) cell lines using the CRISPR/Cas9 genome editing technology. We then selected two clones of A549 KO GL1 (c1 and c3) and two clones of ACHN KO GL1 (cA and cB), in which the total or partial knockout of GABARAPL1 was confirmed by immunoblotting in the presence, or absence, of the inhibitor of autolysosome degradation NH_4_Cl (Figure 3A), as well as by sequencing. Indeed, in the A549 KO GL1 c1, one allele presented the insertion of a G (allele 1), while the other remained wild type (allele 2). Similarly, for the A549 KO GL1 c3, one allele remained wild type (allele 1), while the other presented a deletion of a G (allele 2). For the two ACHN KO GL1 cell lines (cA and cB), no wild-type allele was found, and allele 1 presented an insertion of an A, while allele 2 presented a deletion of an A (Figure S2A). The control cell lines A549c and ACHNc were designed using a CRISPR/Cas9 control plasmid, and were derived from polyclonal cultures. We next confirmed that the knockout of GABARAPL1 was not compensated by the overexpression of the other main ATG8 members—GABARAP, GABARAPL2, and LC3B—at the protein and mRNA levels (Figure S2).

Since GABARAPL1 has been previously described as being involved in the autophagic flux [40,41], we wondered whether the knockout of GABARAPL1 would have an impact on basal autophagy. We then evaluated the levels of LC3B-II via Western blotting experiments. Since the LC3B-II levels are directly correlated with the number of autophagosomes at a particular timepoint [42], it is now widely accepted that an increase in LC3B-II levels can represent either an increase in autophagic induction and vesicle formation, or an inhibition of autolysosome degradation. To study autophagic flux, we therefore compared LC3B-II levels in the presence and absence of NH_4_Cl—an inhibitor of autophagosome/lysosome fusion. Our data showed that the levels of LC3B-II were equivalent between the untreated control cell lines (A549c and ACHNc) and KO cell lines (A549 KO GL1 and ACHN KO GL1). Following NH_4_Cl treatment, we observed an equivalent accumulation of LC3B-II in control and KO GL1 cells (Figure 3B). These results therefore suggest that the knockout of GABARAPL1 has no effect on the basal autophagic flux in A549 and ACHN cells. In order to confirm these results, we used the double-tagged GFP-RFP-LC3 construct and studied the effect of GABARAPL1 knockout on autophagosome and autolysosome numbers [43]. Since GFP fluorescence is sensitive to acidic and proteolytic conditions found in lysosomes, but RFP fluorescence is not, this construct allows for the discrimination of autophagosomes (RFP+/GFP+, yellow staining) and autolysosomes (RFP+/GFP-, red staining). Our data showed that the knockout of GABARAPL1 did not modify the number of autophagosomes and autolysosomes in the different cell lines (Figure 3C), confirming that the knockout of GABARAPL1 did not modify the autophagic flux in A549 cells.

### 3.4. GABARAPL1 Knockout Led to the Induction of EMT

**Figure 4:**
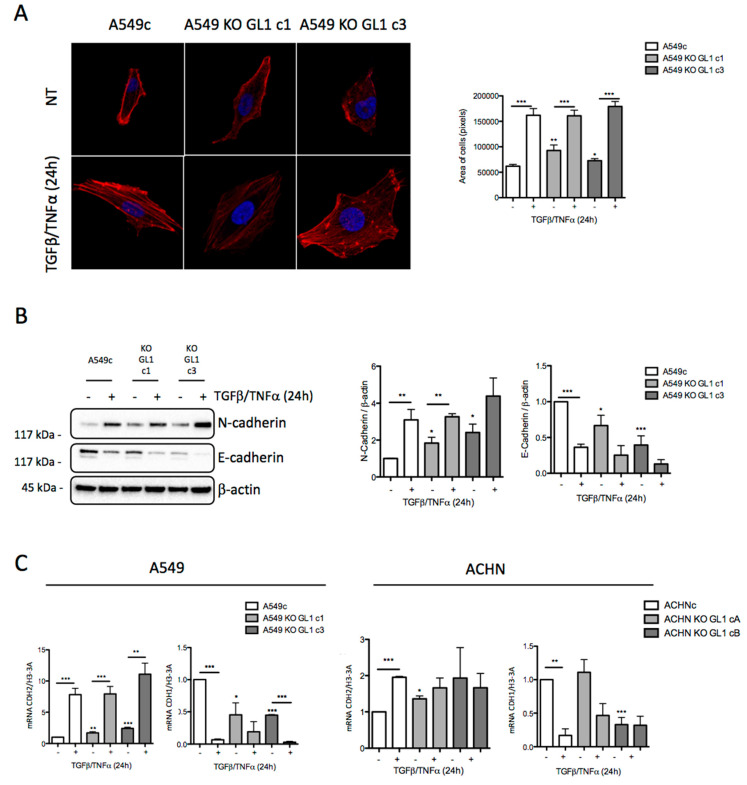
GABARAPL1 knockout leads to an induction of EMT. (**A**) Measurement of the area of A549c and A549 KO GABARAPL1 cells treated, or not, with TGF-β (5 ng/mL) and TNF-α (20 ng/mL) for 24 h. The cells were stained using rhodamine phalloidin, and the cell areas were measured using a confocal microscope and Image J software. Scale bar: 5 μm. Twenty cells were analyzed per condition, and in three independent experiments. (**B**) Western blotting analysis of N-cadherin and E-cadherin levels in A549c and KO GABARAPL1 cells untreated or treated with TGF-β (5 ng/mL) and TNF-α (20 ng/mL) for 24 h. β-actin was used as loading control. Protein levels were quantified using Image Lab. (**C**) mRNA levels of CDH1 and CDH2 were measured by qRT-PCR in A549c and A549 KO GABARAPL1 cells, and in ACHNc and ACHN KO GABARAPL1 cells, untreated or treated with TGF-β 5 ng/mL and TNF-α 20 ng/mL for 24 h. The values calculated by the ∆∆CT method are relative to H3-3A levels, and are expressed as fold change.

We then asked whether GABARAPL1 could regulate EMT. Since, during EMT, cells adopt a fibroblastic spindle-shaped morphology [1], we then analyzed the area of the A549c and KO GABARAPL1 cells, and observed that KO GABARAPL1 cells presented a slightly, but significantly, greater area than the control A549c cells (Figure 4A). Next, we analyzed the expression of two well-described EMT markers: the mesenchymal marker CDH2/N-cadherin, and the epithelial marker CDH1/E-cadherin. Firstly, without treatment, we observed that CDH2/N-cadherin expression was increased, and that CDH1/E-cadherin expression was decreased, in A549 KO GL1 cells compared to A549c cells, suggesting that the KO GL1 cell lines can enter EMT without treatment. After 24 h of treatment with TGF-β/TNF-α, we detected an equivalent increase in the CDH2/N-cadherin levels in A549c and A549 KO GL1 cells. However, a decrease in the CDH1/E-cadherin marker was indeed observed in the A549c cells, as expected, but in a non-significant way in the A549 KO GL1 cells (Figure 4B). Next, we analyzed whether the CDH2/N-cadherin and CDH1/E-cadherin genes were modulated at the mRNA level, and we observed that, in untreated cells, the A549 KO GL1 cells presented an increase in CDH2/N-cadherin mRNA levels, and a decrease in CDH1/E-cadherin mRNA levels, compared to the levels quantified in control A549c cells. We then studied two other well-described EMT markers—the mesenchymal marker vimentin and the epithelial marker EpCAM—in A549 cells, and observed similar results (Figure S3A). Similarly, in ACHN cells, the levels of CDH2/N-cadherin mRNA were increased in the ACHN KO GL1 cells compared to the ACHNc control cells, but in a non-significant way in the ACHN KO GL1 cB cells. Regarding the CDH1/E-cadherin mRNA levels, we showed that they were not modified in ACHN KO GL1 cA cells, but were decreased in the ACHN KO GL1 cB cells. Following a TGF-β/TNF-α treatment, CDH2/N-cadherin mRNA levels were increased while CDH1/E-cadherin mRNA levels were decreased in A549c, A549 KO GL1, and ACHNc, but not in the ACHN KO GL1 cells (Figure 4C). Altogether, these results suggest that the knockout of GABARAPL1 induces EMT through the transcriptional regulation of EMT-related genes. Moreover, the A549 KO GL1 cells, but not the ACHN KO GL1 cells, were still susceptible to the induction of EMT following a TGF-β/TNF-α treatment, even if they were already engaged in EMT without any induction.

### 3.5. GABARAPL1 Knockout Led to an Increase in SNAI1 Levels

**Figure 5:**
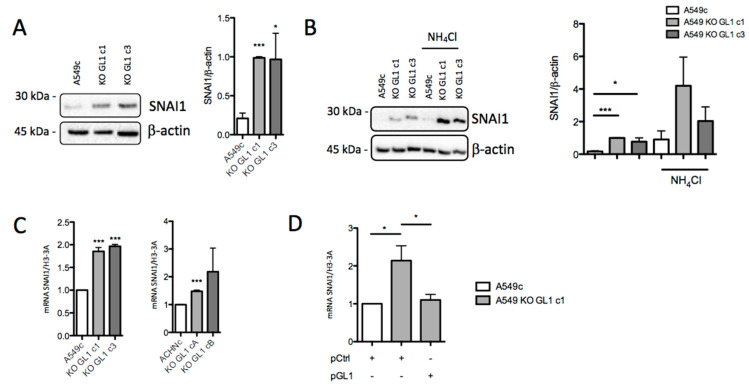
GABARAPL1 knockout leads to an increase in SNAI1 levels. (**A**) Western blotting analysis of SNAI1 levels in untreated A549c and KO GABARAPL1 cells. (**B**) Western blotting analysis of SNAI1 levels in A549c and KO GABARAPL1 cells untreated or treated with 50 mM NH_4_Cl for 2 h. β-actin was used as a loading control. Protein levels were quantified using Image Lab. Data are represented as the mean ± S.E.M. of at least five independent experiments; *p* * < 0.05 compared to the control; *p*-values were calculated using Student’s *t*-test. (**C**) mRNA levels of *SNAI1* were measured by qRT-PCR in untreated A549c, ACHNc, and KO GABARAPL1 cells. The values calculated by the ∆∆CT method are relative to H3-3A levels, and are expressed as fold change. Data are represented as the mean ± S.E.M. of three independent experiments; *p* * < 0.05 compared to the control; *p*-values were calculated using Student’s *t*-test. (**D**) mRNA levels of SNAI1 in A549c cells transfected with pCDNA_Control (pCtrl) or A549 KO GL1 c1 cells transfected either with pCtrl or pCDNA-Flag-GABARAPL1-6His (pGL1). The values calculated by the ∆∆CT method are relative to the levels of H3-3A, and are expressed as fold change.

Since the knockout of GABARAPL1 resulted in an upregulation of the EMT process through the transcriptional downregulation of epithelial markers, such as CDH1/E-cadherin or EpCAM, and the upregulation of mesenchymal markers, such as CDH2/N-cadherin and vimentin, we then hypothesized that GABARAPL1 might regulate the expression or activity of EMT-related transcription factors. In A549 cells, we showed that, in the course of 10–24 h of TGF-β/TNF-α-induced EMT, the EMT-related transcription factor SNAI1 was the gene showing the highest increase in expression, whereas the expression of TWIST and ZEB1 was less induced (Figure S3B). We then analyzed SNAI1 protein levels in A549c and KO GL1 cells, and found that the levels of the SNAI1 protein were greatly increased in A549 KO GL1 cells compared to the A549c control cells (Figure 5A). However, the levels of TWIST were not modified (Figure S3C), suggesting the involvement of SNAI1 in the regulation of the induction of EMT in the A549 cells. The SNAI1 and TWIST transcription factors have been previously shown to be degraded by autophagy in murine hepatocytes, and this degradation may involve selective autophagy, as suggested by the interaction described between SNAI1 and p62/SQSTM1—an autophagy receptor in autophagosomes [31,44]. We therefore wondered whether GABARAPL1 might also induce the selective degradation of SNAI1 by autophagy. To confirm this hypothesis, we analyzed SNAI1 protein levels in A549c in the presence of the inhibitor of autophagosome degradation NH_4_Cl, and we indeed observed an accumulation of SNAI1, confirming that this protein could be degraded by autophagy. However, in A549 KO GL1 cells, the use of NH_4_Cl also led to the same accumulation of SNAI1, suggesting that GABARAPL1 was not directly involved in the degradation of SNAIL by autophagy in A549 cells (Figure 5B). We then analyzed SNAI1 mRNA levels in A549c, ACHNc, and KO GL1 cells. Without any treatment, we found that SNAI1 mRNA levels were increased in A549 and ACHN KO GL1 cells compared to control cells (Figure 5C), whereas the stability of the SNAI1 mRNA was not modified, suggesting a transcriptional regulation of SNAI1 (Figure S3D). We then observed that TGF-β/TNF-α-induced EMT led to an increase in SNAI1 mRNA and protein levels in control and KO GL1 cells (Figure S3E,F), confirming that KO GL1 cells were still inducible in EMT. These results suggest that, in KO GL1 cells, SNAI1 is transcriptionally upregulated, leading to the increase in SNAI1 protein levels, and probably to the increase in CDH2/N-cadherin expression and the decrease in CDH1/E-cadherin expression. As expected, overexpression of GABARAPL1 by transient transfection of pGL1 in the A549 KO GABARAPL1 cells decreased SNAI1 expression compared to pCtrl-transfected cells (Figure 5D).

### 3.6. GABARAPL1 Induced the Degradation of SMAD by Autophagy

**Figure 6:**
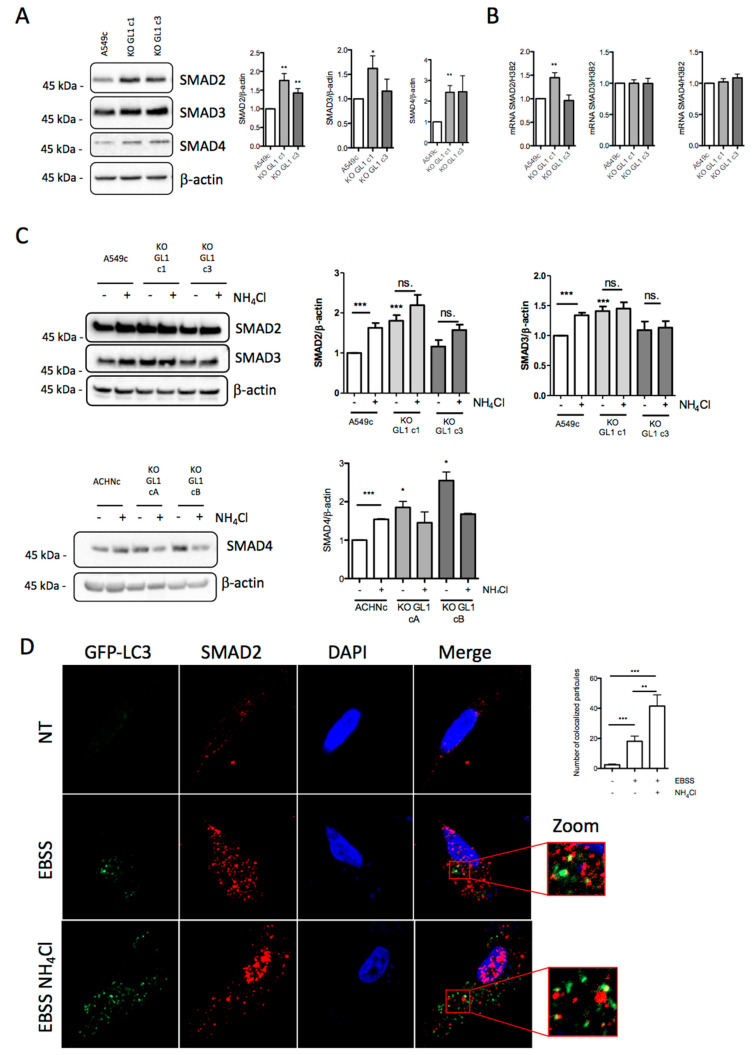

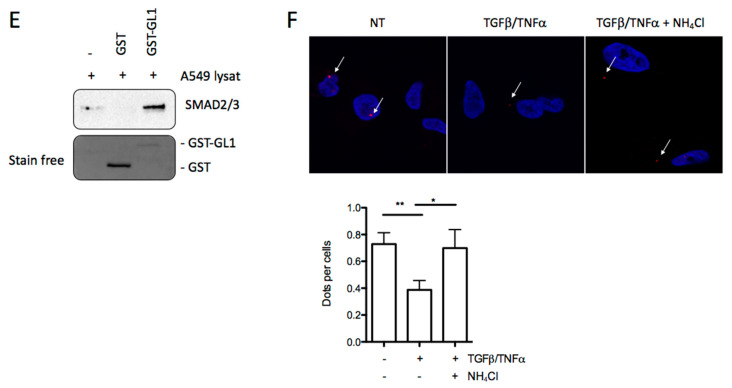
GABARAPL1 knockout leads to an increase in SMAD protein levels by inhibiting their autophagic degradation. (**A**) Western blotting analysis of SMAD2, SMAD3, and SMAD4 levels in untreated A549c and KO GABARAPL1 cells. (**B**) mRNA levels of SMAD2, SMAD3, and SMAD4 were measured by qRT-PCR in untreated A549c and KO GABARAPL1 cells. The values calculated by the ∆∆CT method are relative to H3-3A levels, and are expressed as fold change. (**C**) Western blotting analysis of SMAD2 and SMAD3 in A549c and KO GABARAPL1, and of SMAD4 in ACHNc and KO GABARAPL1 cells, untreated or treated with 50 mM NH_4_Cl for 2 h. β-actin was used as a loading control. Protein levels were quantified using Image Lab. (**D**) Colocalization of LC3 and SMAD2 in A549c cells transfected with pGFP-LC3 vector and immunostained for SMAD2. Colocalization of the autophagosome markers GFP-LC3 and SMAD2 was analyzed using a confocal microscope and the object base method using the “JACoP” plugin (ImageJ software). Scale bar: 5 μm. (**E**) GST pull-down analysis of the interaction between GABARAPL1 and SMAD2/3 proteins in A549c whole-cell lysates. Whole A549c cell lysate was submitted in a GST pull-down assay with either GST-GABARAPL1 or GST- as a negative control. Data are representative of three independent experiments. (**F**) P-LISA analysis of GABARAPL1–SMAD2/3 interaction (red) in A549c cells untreated or treated with TGF-β 5 ng/mL and TNF-α 20 ng/mL for 24 h in the presence or absence of 50 mM NH_4_Cl for 2 h. A representative image of three independent experiments is shown. The number of red dots per cell was quantified in at least 30 randomly selected cells in each experiment. Scale bar: 5 μm.

Since the knockout of GABARAPL1 led to an increase in SNAI1 expression, we analyzed the SMAD signaling pathway, which has been extensively described as being involved in SNAI1 transcription regulation [4]. We therefore decided to study SMAD2, SMAD3, and SMAD4 basal protein levels, and found that their levels were increased in A549 KO GL1 cells compared to A549c cells (Figure 6A). Even if the increase in SMAD protein levels was not correlated with mRNA levels, we still detected a slight increase in SMAD2 mRNA levels, but only in the A549 KO GL1 c1 cells (Figure 6B). Similarly, we found that SMAD4 was increased in ACHN KO GL1 cells compared to ACHNc cells, but no differences in SMAD2 and SMAD3 levels were detected (Figure 6C and Figure S4A). We therefore hypothesized that this increase in SMAD protein levels could explain the increase in SNAI1 mRNA levels. We then hypothesized that the increase in SMAD protein levels in A549 and ACHN KO GL1 cells may be explained by a decrease in their degradation by autophagy. To address this hypothesis, we inhibited autophagic degradation and quantified SMAD2 and SMAD3 levels in control A549c cells, and found that the NH_4_Cl treatment led to an accumulation of SMAD2 and SMAD3 in control A549c cells, but not in A549 KO GL1 cells (Figure 6C). Similarly, the NH_4_Cl treatment led to an accumulation of SMAD4 in control ACHNc cells, but not in ACHN KO GL1 cells. We then wondered whether the SMAD proteins could be localized in the autophagosomes. To find out, we studied the colocalization between autophagosomes (GFP-LC3, green) and SMAD2 (red). We did not detect any autophagosomes (absence of puncta) without treatment, but when we induced the autophagy with EBSS, we showed found SMAD2 proteins could indeed be localized in autophagosomes. Moreover, when we blocked the autophagic degradation using NH_4_Cl, we observed an increase in the colocalization between SMAD2 and GFP-LC3 (Figure 6D)—probably due to the increase in the number of autophagosomes analyzed (Figure S4B). Altogether, these results suggest an inhibition of the degradation of SMAD2 and SMAD3 by autophagy in A549 KO GL1 cells, and an inhibition of the degradation of SMAD4 by autophagy in ACHN KO GL1 cells.

Since we observed a decrease in the degradation of SMADs by autophagy in the KO GL1 cells without any modification of their autophagic flux, we then hypothesized that SMADs may be degraded through GABARAPL1-mediated selective autophagy. This hypothesis was corroborated by the analysis of the amino acid sequences of SMADs using the iLIR autophagy database, which showed that SMAD2 contains seven potential AIM sequences, and that SMAD3 and SMAD4 contain eight potential AIM sequences each [45] (Figure S4C). We therefore tested the interaction between GABARAPL1 and SMAD proteins using an in vitro GST pull-down assay using A549 whole-cell lysates, and found that GABARAPL1 could indeed interact with SMAD2/3 proteins (Figure 6E). These results were confirmed with an in silico proximity ligation assay, which showed that GABARAPL1 could indeed interact with SMAD2/3 in untreated A549c cells (Figure 6F). Then, the TGF-β/TNF-α treatment led to a decrease in the GABARAPL1–SMAD2/3 interaction due to the activation of the autophagic flux in this condition (Figure S4D) and, therefore, the degradation of the SMAD and GABARAPL1 proteins. As expected, the inhibition of the autophagic flux using NH_4_Cl restored the number of GABARAPL1–SMAD2/3 interactions (Figure 6F).

### 3.7. GABARAPL1 Inhibited Aggressive Cancer Phenotypes

**Figure 7:**
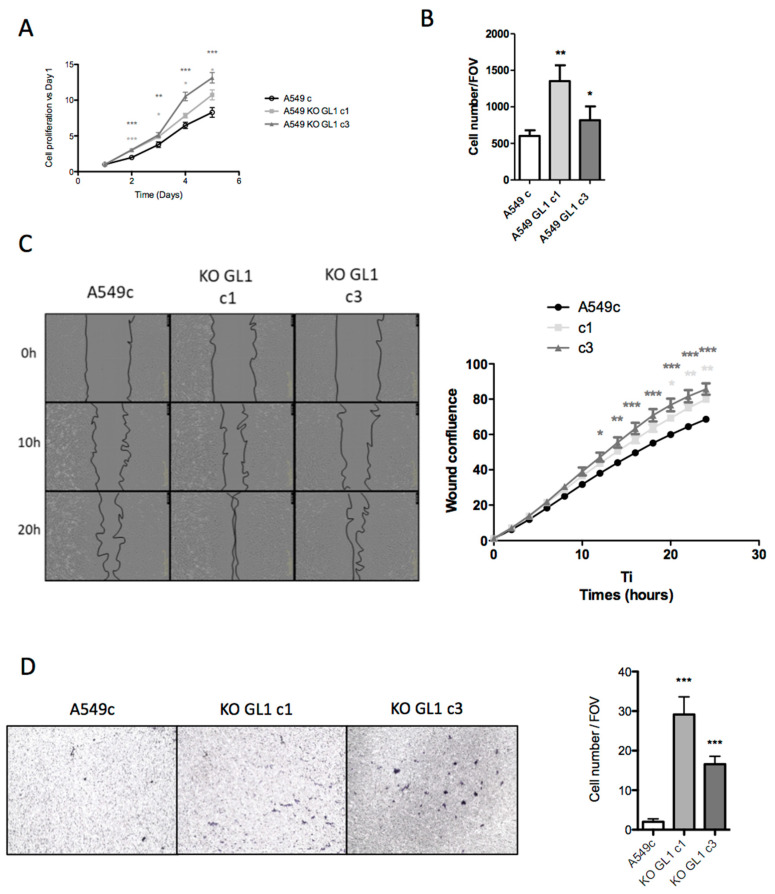
GABARAPL1 knockout increases cell proliferation and invasion. (**A**) Growth rate of A549c and KO GABARAPL1 cells using MTT assay. The data shown are representative of three independent experiments performed in eight replicates. (**B**) Invasion of A549c, KO GL1 c1 and KO GL1 c3 in modified Boyden chambers. Cell density was estimated via photographs taken using an EVOS XL Core microscope. (**C**) Migration rates of A549c, KO GL1 c1, and KO GL1 c3 using a scratch wound-healing assay. Cell migration was quantified by the confluence wound study using IncuCyte from 0 to 24 h. (**D**) Migration rates of A549c, KO GL1 c1, and KO GL1 c3 using modified Boyden chambers. A representative image of each membrane is shown. Six FOVs were randomly selected, and the number of cells that migrated through the membrane was determined.

Since GABARAPL1 has been previously described as a tumor suppressor gene in breast cancer and hepatocellular carcinoma cell lines [46,47], we therefore studied the effect of the knockout of GABARAPL1 in our lung adenocarcinoma cell line A549, and found that the A549 KO GABARAPL1 cells presented a higher proliferation rate than the control A549c cells (Figure 7A).

During EMT, cancer cells acquire mobility properties; we thus wondered about the impact of the knockout of GABARAPL1 on invasiveness and migration. As expected, we also found that the A549 KO GABARAPL1 cells presented higher migration and invasion rates than the control A549c cells (Figure 7B–D). These results could be explained by the decrease in the E-cadherin/CDH1 levels involved in cellular junction and tissue organization, as well as the increase in the N-cadherin/CDH2 levels involved in cell mobility.

Taken together, we proposed a model describing how the expression of GABARAPL1 was increased in the EMT+ tumors through the activation of the SMAD signaling pathway. Moreover, we found that GABARAPL1 negatively regulated EMT through its involvement in the selective degradation of the SMAD proteins, leading to the inhibition of the migratory and invasive abilities of the cancer cells.

## 4. Discussion

Autophagy is a catabolic intracellular process that can induce the random elimination of sections of cytoplasm to feed nutrients to the cancer cells in order to sustain their hyperactive metabolism [27]. Nevertheless, a specialized type of autophagy—called selective autophagy—involving the ATG8 proteins can lead to the targeted elimination of cell components [48]. Indeed, selective autophagy has been described as being involved in the regulation of EMT through the degradation of EMT factors such as SNAI1.

ATG8 gene expression has been poorly studied in cancers and during EMT [20]. In our study, we found that ATG8 expression is correlated with EMT markers, and that GABARAPL1 is the gene most correlated with the EMT markers in lung adenocarcinoma tumors. We also found that the most aggressive EMT+ adenocarcinoma tumors presented higher GABARAPL1 protein levels compared to the epithelial tumors. Our results are in agreement with previous works showing that, in gastric cancers, LC3B expression is correlated with the expression of the EMT marker vimentin, and associated with poor clinical outcomes [49]. Our observations have also been confirmed in A549 lung adenocarcinoma cells and in MCF10A immortalized breast cells with induced EMT, where ATG8 gene expression was modulated during EMT, but amongst them, GABARAPL1 appeared to be the most highly expressed gene. However, in the ACHN kidney adenocarcinoma cells, we only observed an increase in LC3B expression. These results might be explained by the fact that A549 and MCF10A cells present low basal levels of GABARAPL1 compared to the untreated ACHN cells, which present higher basal levels of GABARAPL1, suggesting that its expression might not be capable of further increase. We also observed that the TGF-β/TNF-α treatment induced autophagic flux in the A549 cells, and that the increase in GABARAPL1 expression was regulated by the SMAD signaling pathway in these cells. Our results are in agreement with previous studies showing that TGF-β induces the expression of the autophagy-related genes BECN1, ATG5, and ATG7 through the SMAD signaling pathway in hepatocellular cancer cells, leading to the activation of autophagy [50].

In our study, we demonstrated that GABARAPL1-mediated selective autophagy might be involved in the degradation of the SMAD proteins, leading to decreased SNAI1 expression, the inhibition of EMT signaling, and the decrease in EMT-associated phenotypes. We also demonstrated that autophagy can degrade SNAI1, but in a GABARAPL1-independent manner. These results are in agreement with previous works showing that autophagy is involved in the degradation of the TGF-β, TWIST, and NBR1 proteins, and that the ATG8 proteins can act as tumor suppressors. Indeed, the expression of GABARAP family members has been described as most often downregulated in cancers, and their high expression has been linked to a favorable prognosis in several cancer types. In vitro studies have shown that this is mostly due to their involvement in the selective degradation of several oncogenic proteins, as described for the LC3-mediated degradation of SNAI1 [20,30,51,52]. However, the ATG8 proteins might also be involved in cancer inhibition in a selective-autophagy-independent pathway, via the regulation of several signaling pathways involved in cancer progression [40,53].

We demonstrated, for the first time, that the decrease in SNAI1 levels and the consequent inhibition of EMT signaling linked to the autophagic activity is not only due to the selective degradation of the SNAI1 protein by autophagy, but could also be linked to the degradation of the transcription factor SMAD by autophagy, and perhaps by GABARAPL1-mediated autophagy, which would lead to a decrease in SNAI1 transcription.

The SMAD2/SMAD3/SMAD4 signaling pathway has been previously described as being the main driver of TGF-β-induced EMT. In our model, this pathway specifically induced the expression of SNAI1, which has been described as acting as both a transcriptional repressor of epithelial genes—such as CDH1/E-cadherin—and a transcriptional inducer of mesenchymal genes, such as CDH2/N-cadherin [54]. These observations might explain why the knockout of GABARAPL1 increased SNAI1 protein levels, but did not modify the TWIST protein levels. Then, we showed that SMAD2 was detected in the autophagosomes, and that GABARAPL1 interacted with SMAD2/3 proteins in cellulo. This interaction appeared to be regulated by the TGF-β/TNF-α treatment, leading to the induction of EMT, but also to the induction of autophagy. Therefore, the TGF-β/TNF-α-induced autophagy led to the degradation of both GABARAPL1 and the SMAD proteins, explaining the decrease in GABARAPL1–SMAD interaction. Moreover, the inhibition of the autophagic degradation restored the initial levels of interaction between the GABARAPL1 and SMAD2/3 proteins.

Our results are supported by an in silico analysis showing that SMAD proteins contain multiple potential AIM (ATG8-interacting motif) sequences described as being necessary for recruitment into the autophagosomes and degradation by the ATG8 proteins [45,55], leading to the inhibition of the SMAD signaling pathway and, therefore, EMT. The involvement of GABARAPL1 in the degradation of SMAD explains why the knockout of GABARAPL1 increased SMAD protein levels, SNAI1 transcription and, therefore, EMT. Surprisingly, in the A549 cells, we observed an increase in SMAD2, SMAD3, and SMAD4 protein levels, while in the ACHN cells, we only detected an increase in SMAD4 levels. These observations indicate that some ATG8s may specifically recruit proteins, depending on the tumor type. These observations might reflect the existing balance between the specialization and the functional redundancy of the ATG8 proteins [20]. The specialization of the ATG8 proteins is supported by the differences existing in their amino acid sequences around their AIM, described as being involved in the specificity of the interaction between ATG8s and their different partners [56]. However, it has also been suggested that the ATG8 proteins could have common partners, such as ULK1 [18,57].

The specificity of the involvement of GABARAPL1 in the selective degradation of SMAD by autophagy is also supported by the fact that, in our models, the knockout of GABARAPL1 did not modify the autophagic flux and, thus, probably did not modify the non-selective degradation of proteins by autophagy. Nevertheless, our team has previously shown that, in breast cancer cell lines, GABARAPL1 is involved in autophagic flux [40,41]. These contradictory results might be attributable to the GABARAP family proteins performing redundant roles in basal autophagic flux [58], and the high expression of GABARAP in our model might functionally compensate for the loss for GABARAPL1 during basal autophagic flux.

The degradation of SMADs by the selective autophagy process might be a new EMT-negative regulatory loop. Indeed, GABARAPL1 expression is increased during the induction of EMT through the SMAD signaling pathway, as has been previously described for BECN1, ATG5, and ATG7 [50], and this increase in GABARAPL1 protein levels might lead to the inhibition of EMT through the degradation of SMAD. It would be interesting to study the precise negative feedback function of GABARAPL1 during EMT: does GABARAPL1 inhibit EMT, or can it also initiate the reverse process—a mesenchymal–epithelial transition (MET)?

In this study, we showed for the first time that GABARAPL1 expression was correlated with EMT markers in human lung adenocarcinoma tumors, and might intervene in an EMT-negative regulatory loop. Indeed, the induction of EMT led to the increase in GABARAPL1 levels through the activation of the SMAD signaling pathway, and GABARAPL1 then induced the selective degradation of SMAD by autophagy, therefore leading to the decrease in SNAI1 transcription and the inhibition of EMT (Figure S5).

## Supplementary Materials

The following are available online at https://www.mdpi.com/article/10.3390/biology10100956/s1, Figures S1–S4. MCF10A (immortalized mammary epithelial cells) and ACHN (kidney adenocarcinoma, ATCC CRL-1611) cancer cells were cultured in DMEM 1g/L glucose (L0066, Dominique Dutscher) supplemented with 10% FBS (702774C, Corning) and 100 µg/mL of penicillin/streptomycin (PAA, P11-010) in a 5% CO_2_ incubator at 37 °C. To study mRNA stability, cells were incubated with 2.5 µg/mL of actinomycin D (EU0450-B, Euromedex) from 15 min to 4 h. For the Western blotting experiment, the following antibodies were used: GABARAP/L1 (AB15278, Chemicon Millipore), GABARAPL2 (18724-1-AP, ProteinTech), and TWIST1/2 (GTX127310, GeneTex). For qPCR, the following primers were used: TWIST (F-CGGGAGTCCGCAGTCTTA and R-TGAATCTTGCTCAGCTTGTC) and ZEB1 (F- AGCAGTGAAAGAGAAGGGAATGC and R-GGTCCTCTTCAGGTGCCTCAG).

## References

[1] Brabletz T., Kalluri R., Nieto M.A., Weinberg R.A. (2018). EMT in cancer. Nat. Rev. Cancer.

[2] Massagué J. (2008). TGFβ in Cancer. Cell.

[3] Nieto M.A., Huang R.Y.-J., Jackson R.A., Thiery J.P. (2016). EMT: 2016. Cell.

[4] Ahmadi A., Najafi M., Farhood B., Mortezaee K. (2018). Transforming growth factor-β signaling: Tumorigenesis and targeting for cancer therapy. J. Cell. Physiol..

[5] Rubinsztein D.C., Shpilka T., Elazar Z. (2012). Mechanisms of Autophagosome Biogenesis. Curr. Biol..

[6] Yang Z., Klionsky D.J. (2010). Mammalian autophagy: Core molecular machinery and signaling regulation. Curr. Opin. Cell Biol..

[7] Ashford T.P., Porter K.R. (1962). Cytoplasmic Components in Hepatic Cell Lysosomes. J. Cell Biol..

[8] Fortun J., Dunn W.A., Joy S., Li J., Notterpek L. (2003). Emerging Role for Autophagy in the Removal of Aggresomes in Schwann Cells. J. Neurosci..

[9] Lamark T., Johansen T. (2012). Aggrephagy: Selective Disposal of Protein Aggregates by Macroautophagy. Int. J. Cell Biol..

[10] He H., Dang Y., Dai F., Guo Z., Wu J., She X., Pei Y., Chen Y., Ling W., Wu C. (2003). Post-translational Modifications of Three Members of the Human MAP1LC3 Family and Detection of a Novel Type of Modification for MAP1LC3B. J. Biol. Chem..

[11] Legesse-Miller A., Sagiv Y., Porat A., Elazar Z. (1998). Isolation and Characterization of a Novel Low Molecular Weight Protein Involved in Intra-Golgi Traffic. J. Biol. Chem..

[12] Mann S., Hammarback J. (1994). Molecular characterization of light chain 3. A microtubule binding subunit of MAP1A and MAP1B. J. Biol. Chem..

[13] Mansuy V., Boireau W., Fraichard A., Schlick J.-L., Jouvenot M., Delage-Mourroux R. (2004). GEC1, a protein related to GABARAP, interacts with tubulin and GABA(A) receptor. Biochem. Biophys. Res. Commun..

[14] Pellerin I., Vuillermoz C., Jouvenot M., Ordener C., Royez M., Adessi G.L. (1993). Identification and characterization of an early estrogen-regulated RNA in cultured guinea-pig endometrial cells. Mol. Cell. Endocrinol..

[15] Sagiv Y., Legesse-Miller A., Porat A., Elazar Z. (2000). GATE-16, a membrane transport modulator, interacts with NSF and the Golgi v-SNARE GOS-28. EMBO J..

[16] Wang H., Bedford F.K., Brandon N.J., Moss S.J., Olsen R.W. (1999). GABA(A)-receptor-associated protein links GABA(A) receptors and the cytoskeleton. Nature.

[17] Xin Y., Yu L., Chen Z., Zheng L., Fu Q., Jiang J., Zhang P., Gong R., Zhao S. (2001). Cloning, Expression Patterns, and Chromosome Localization of Three Human and Two Mouse Homologues of GABAA Receptor-Associated Protein. Genomics.

[18] Alemu E.A., Lamark T., Torgersen K.M., Birgisdottir A.B., Larsen K.B., Jain A., Olsvik H., Øvervatn A., Kirkin V., Johansen T. (2012). ATG8 Family Proteins Act as Scaffolds for Assembly of the ULK Complex: Sequence requirements for LC3-interacting region (LIR) motifs. J. Biol. Chem..

[19] Rogov V.V., Stolz A., Ravichandran A.C., Rios-Szwed D., Suzuki H., Kniss A., Löhr F., Wakatsuki S., Dötsch V., Dikic I. (2017). Structural and functional analysis of the GABARAP interaction motif (GIM). EMBO Rep..

[20] Jacquet M., Guittaut M., Fraichard A., Despouy G. (2020). The function of ATG8 proteins in autophagy and cancer: Linked or unrelated?. Autophagy.

[21] Murray D., Mirzayans R., McBride W.H. (2018). Defenses against Pro-oxidant Forces—Maintenance of Cellular and Genomic Integrity and Longevity. Radiat. Res..

[22] Goussetis D.J., Gounaris E., Wu E.J., Vakana E., Sharma B., Bogyo M., Altman J.K., Platanias L.C. (2012). Autophagic degradation of the BCR-ABL oncoprotein and generation of antileukemic responses by arsenic trioxide. Blood.

[23] Belaid A., Cerezo M., Chargui A., Corcelle-Termeau E., Pedeutour F., Giuliano S., Ilie M., Rubera I., Tauc M., Barale S. (2013). Autophagy Plays a Critical Role in the Degradation of Active RHOA, the Control of Cell Cytokinesis, and Genomic Stability. Cancer Res..

[24] Elgendy M., Sheridan C., Brumatti G., Martin S.J. (2011). Oncogenic Ras-induced expression of Noxa and Beclin-1 promotes autophagic cell death and limits clonogenic survival. Mol. Cell..

[25] Yu L., Wan F., Dutta S., Welsh S., Liu Z., Freundt E., Baehrecke E.H., Lenardo M. (2006). Autophagic programmed cell death by selective catalase degradation. Proc. Natl. Acad. Sci. USA.

[26] Liu H., He Z., von Rütte T., Yousefi S., Hunger R.E., Simon H.-U. (2013). Down-Regulation of Autophagy-Related Protein 5 (ATG5) Contributes to the Pathogenesis of Early-Stage Cutaneous Melanoma. Sci. Transl. Med..

[27] Folkerts H., Hilgendorf S., Vellenga E., Bremer E., Wiersma V.R. (2018). The multifaceted role of autophagy in cancer and the microenvironment. Med. Res. Rev..

[28] Chen Y., Sun H.-Q., Eichorst J.P., Albanesi J.P., Yin H., Mueller J.D. (2018). Co-mobility of GABARAP and Phosphatidylinositol 4-kinase 2A on cytoplasmic vesicles. Biochemistry.

[29] Sharif T., Martell E., Dai C., Kennedy B.E., Murphy P., Clements D.R., Kim Y., Lee P.W.K., Gujar S.A. (2016). Autophagic homeostasis is required for the pluripotency of cancer stem cells. Autophagy.

[30] Catalano M., D’Alessandro G., Lepore F., Corazzari M., Caldarola S., Valacca C., Faienza F., Esposito V., Limatola C., Cecconi F. (2015). Autophagy induction impairs migration and invasion by reversing EMT in glioblastoma cells. Mol. Oncol..

[31] Grassi G., Di Caprio G., Santangelo L., Fimia G.M., Cozzolino A.M., Komatsu M., Ippolito G., Tripodi M., Alonzi T. (2015). Autophagy regulates hepatocyte identity and epithelial-to-mesenchymal and mesenchymal-to-epithelial transitions promoting Snail degradation. Cell Death Dis..

[32] Lv Q., Wang W., Xue J., Hua F., Mu R., Lin H., Yan J., Lv X., Chen X., Hu Z.-W. (2012). DEDD Interacts with PI3KC3 to Activate Autophagy and Attenuate Epithelial–Mesenchymal Transition in Human Breast Cancer. Cancer Res..

[33] Yang Z., Bian E., Xu Y., Ji X., Tang F., Ma C., Wang H., Zhao B. (2020). Meg3 Induces EMT and Invasion of Glioma Cells via Autophagy. OncoTargets Ther..

[34] Tong H., Yin H., Hossain M.A., Wang Y., Wu F., Dong X., Gao S., Zhan K., He W. (2018). Starvation-induced autophagy promotes the invasion and migration of human bladder cancer cells via TGF-β1/Smad3-mediated epithelial-mesenchymal transition activation. J. Cell Biochem..

[35] Zou M., Zhu W., Wang L., Shi L., Gao R., Ou Y., Chen X., Wang Z., Jiang A., Liu K. (2016). AEG-1/MTDH-activated autophagy enhances human malignant glioma susceptibility to TGF-β1-triggered epithelial-mesenchymal transition. Oncotarget.

[36] Herfs M., Longuespée R., Quick C.M., Roncarati P., Suarez-Carmona M., Hubert P., Lebeau A., Bruyere D., Mazzucchelli G., Smargiasso N. (2016). Proteomic signatures reveal a dualistic and clinically relevant classification of anal canal carcinoma. J. Pathol..

[37] Hubert P., Herman L., Roncarati P., Maillard C., Renoux V., Demoulin S., Erpicum C., Foidart J.-M., Boniver J., Noel A. (2014). Altered α-defensin 5 expression in cervical squamocolumnar junction: Implication in the formation of a viral/tumour-permissive microenvironment. J. Pathol..

[38] Gauthier T., Claude-Taupin A., Delage-Mourroux R., Boyer-Guittaut M., Hervouet E. (2015). Proximity Ligation In situ Assay is a Powerful Tool to Monitor Specific ATG Protein Interactions following Autophagy Induction. PLoS ONE.

[39] Peixoto P., Etcheverry A., Aubry M., Missey A., Lachat C., Perrard J., Hendrick E., Delage-Mourroux R., Mosser J., Borg C. (2019). EMT is associated with an epigenetic signature of ECM remodeling genes. Cell Death Dis..

[40] Poillet-Perez L., Jacquet M., Hervouet E., Gauthier T., Fraichard A., Borg C., Pallandre J.-R., Gonzalez B.J., Ramdani Y., Boyer-Guittaut M. (2017). GABARAPL1 tumor suppressive function is independent of its conjugation to autophagosomes in MCF-7 breast cancer cells. Oncotarget.

[41] Boyer-Guittaut M., Poillet L., Liang Q., Bôle-Richard E., Ouyang X., Benavides G.A., Chakrama F.Z., Fraichard A., Darley-Usmar V.M., Despouy G. (2014). The role of GABARAPL1/GEC1 in autophagic flux and mitochondrial quality control in MDA-MB-436 breast cancer cells. Autophagy.

[42] Tanida I., Ueno T., Kominami E. (2004). Human Light Chain 3/MAP1LC3B Is Cleaved at Its Carboxyl-terminal Met121 to Expose Gly120 for Lipidation and Targeting to Autophagosomal Membranes. J. Biol. Chem..

[43] Kimura S., Noda T., Yoshimori T. (2007). Dissection of the Autophagosome Maturation Process by a Novel Reporter Protein, Tandem Fluorescent-Tagged LC3. Autophagy.

[44] Pankiv S., Clausen T.H., Lamark T., Brech A., Bruun J.A., Outzen H., Øvervatn A., Bjørkøy G., Johansen T. (2007). p62/SQSTM1 binds directly to Atg8/LC3 to facilitate degradation of ubiquitinated protein aggregates by autophagy. J. Biol. Chem..

[45] Jacomin A.-C., Samavedam S., Promponas V., Nezis I.P. (2016). iLIR database: A web resource for LIR motif-containing proteins in eukaryotes. Autophagy.

[46] Berthier A., Seguin S., Sasco A.J., Bobin J.Y., De Laroche G., Datchary J., Saez S., Rodriguez-Lafrasse C., Tolle F., Fraichard A. (2010). High expression of gabarapl1 is associated with a better outcome for patients with lymph node-positive breast cancer. Br. J. Cancer.

[47] Zhang Y., Wang F., Han L., Wu Y., Li S., Yang X., Wang Y., Ren F., Zhai Y., Wang D. (2011). GABARAPL1 Negatively Regulates Wnt/β-catenin Signaling by Mediating Dvl2 Degradation through the Autophagy Pathway. Cell. Physiol. Biochem..

[48] Anding A.L., Baehrecke E.H. (2017). Cleaning House: Selective Autophagy of Organelles. Dev. Cell.

[49] Wang J.-Y., Wu T., Ma W., Li S., Jing W.-J., Ma J., Chen D.-M., Guo X.-J., Nan K.-J. (2018). Expression and clinical significance of autophagic protein LC3B and EMT markers in gastric cancer. Cancer Manag. Res..

[50] Kiyono K., Suzuki H.I., Matsuyama H., Morishita Y., Komuro A., Kano M., Sugimoto K., Miyazono K. (2009). Autophagy Is Activated by TGF-β and Potentiates TGF-β–Mediated Growth Inhibition in Human Hepatocellular Carcinoma Cells. Cancer Res..

[51] Zada S., Hwang J.S., Ahmed M., Lay T.H., Pham T.M., Kim D.R. (2019). Control of the Epithelial-to-Mesenchymal Transition and Cancer Metastasis by Autophagy-Dependent SNAI1 Degradation. Cells.

[52] Marsh T., Debnath J. (2020). Autophagy suppresses breast cancer metastasis by degrading NBR1. Autophagy.

[53] Liu Y., Wang D., Lei M., Gao J., Cui Y., Jin X., Yu Q., Jiang Y., Guo Y., Liu Y. (2021). GABARAP suppresses EMT and breast cancer progression via the AKT/mTOR signaling pathway. Aging.

[54] Xu J., Lamouille S., Derynck R. (2009). TGF-β-induced epithelial to mesenchymal transition. Cell Res..

[55] Birgisdottir A.B., Lamark T., Johansen T. (2013). The LIR motif—Crucial for selective autophagy. J. Cell Sci..

[56] Atkinson J.M., Ye Y., Gebru M.T., Liu Q., Zhou S., Young M.M., Takahashi Y., Lin Q., Tian F., Wang H.-G. (2019). Time-resolved FRET and NMR analyses reveal selective binding of peptides containing the LC3-interacting region to ATG8 family proteins. J. Biol. Chem..

[57] Joachim J., Jefferies H.B.J., Razi M., Frith D., Snijders A.P., Chakravarty P., Judith D., Tooze S.A. (2015). Activation of ULK Kinase and Autophagy by GABARAP Trafficking from the Centrosome Is Regulated by WAC and GM130. Mol. Cell..

[58] Nguyen T.N., Padman B.S., Usher J., Oorschot V., Ramm G., Lazarou M. (2016). Atg8 family LC3/GABARAP proteins are crucial for autophagosome-lysosome fusion but not autophagosome formation during PINK1/Parkin mitophagy and starvation. J. Cell Biol..

