# Supplementary material for: GABARAPL1 Inhibits EMT Signaling through SMAD-Tageted Negative Feedback
Source: Biology (Basel). 2021 Sep 24;10(10):956. doi: 10.3390/biology10100956 (PMC8533302; doi:10.3390/biology10100956)
Supplement: Supplementary file 1 [file biology-10-00956-s001.zip › supplem.pdf]

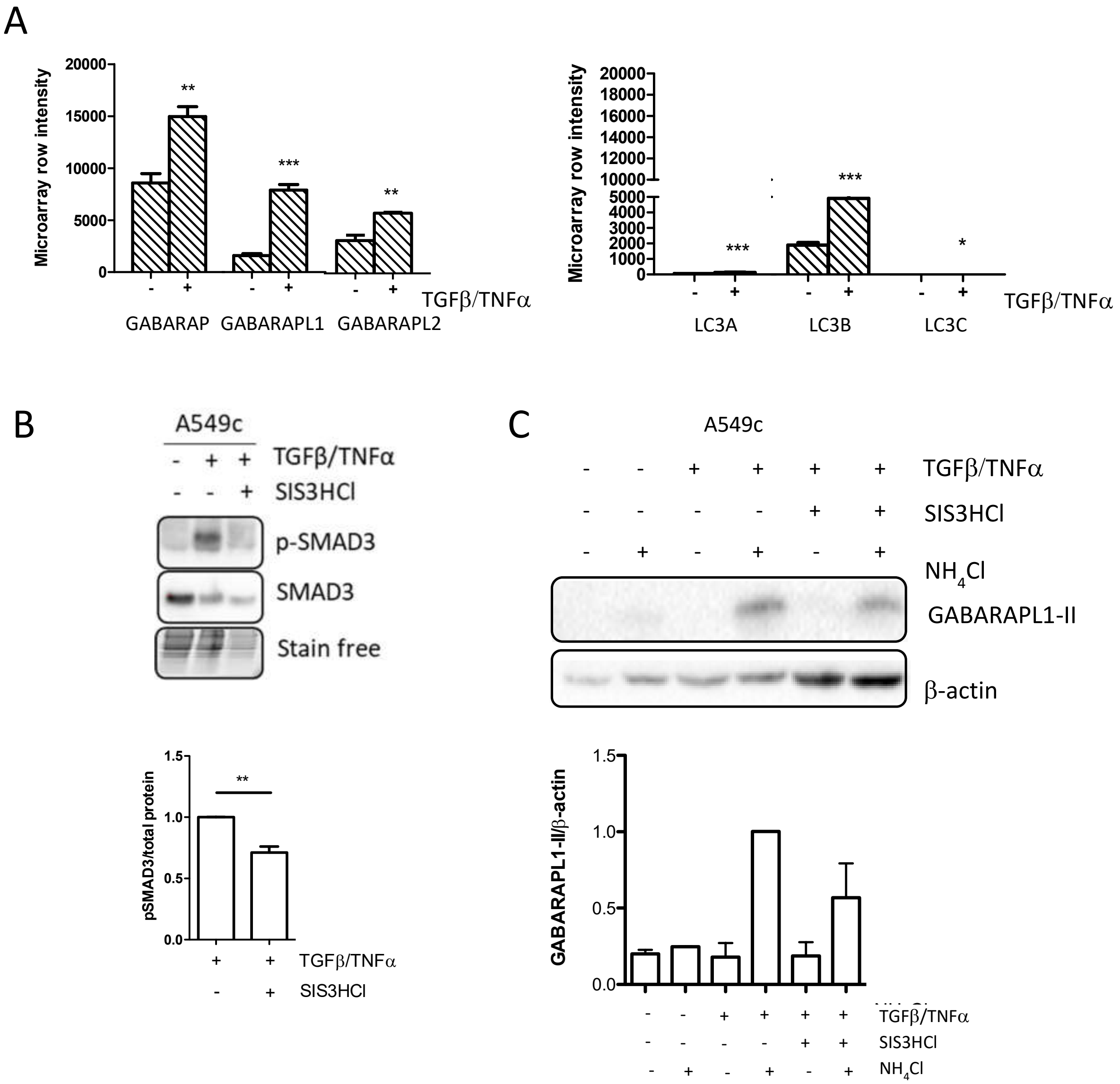

Figure S1.

(A) Transcriptome analysis of ATG8 gene expression. ATG8 gene expression was quantified by microarray in MCF10A cells untreated or treated with TGFβ (5 ng/ml) and TNFα (20 ng/ml) for 5 days (26). Data are represented as ±S.E.M. of four independent experiments. P*<0,05 compared to the control ; P-values were calculated using a Student's t test. (B) Western blot analysis of p-SMAD3 and SMAD3 in A549c cells. Cells have been pretreated or not by 20 μM SIS3HCl for 6 h and then treated by with TGFβ (5 ng/ml) and TNFα (20 ng/ml) for 24 h. β-actin was used as a loading control. Protein levels were quantified using Image Lab. Data are represented as ±S.E.M. of at three independent experiments. P*<0,05 compared to the control ; P-values were calculated using a Student's t test. (C) Western blot analysis of GABARAPL1-II in A549c cells. Cells have been pretreated or not by 20 μM SIS3HCl for 6 h and then treated by with TGFβ (5 ng/ml) and TNFα (20 ng/ml) for 24 h in presence or in absence of 50 mM NH4Cl. β-actin was used as a loading control. Protein levels were quantified using Image Lab. Data are represented as ±S.E.M. of two independent experiments. P*<0,05 compared to the control ; P-values were calculated using a Student's t test.

A

Sample	Start	Sequence	End
ADNg GABARAPL1 A549c :	245	CGGTGCAATCATGAAGTTCCAGTAACAAGGAGGACCATCCCTTTGAG	289
ADNg GABARAPL1 A549 KO GL1 c1 (All 1):		CGGTGCAATCATGAAGTTCCAGTAACAAGGAGGACCATCCCTTTGAG	
ADNg GABARAPL1 A549 KO GL1 c1 (All 2):		CGGTGCAATCATGAAGTTCCAGTAACAAGGAGGACCATCCCTTTGAG	
ADNg GABARAPL1 A549 KO GL1 c3 (All 1):		CGGTGCAATCATGAAGTTCCAGTAACAAGGAGGACCATCCCTTTGAG	
ADNg GABARAPL1 A549 KO GL1 c3 (All 2):		CGGTGCAATCATGAAGTTCCAGTAACAAGGAGGACCATCCCTTTGA**-**T	

Sample	Start	Sequence	End
ADNg GABARAPL1 A549c :	290	TATCGGAAAAAGGAAGGAGAAAAGATCCGGAAGAAATATCCGGACA	335
ADNg GABARAPL1 A549 KO GL1 c1 (All 1):		TATCGGAAAAAGGAAGGAGAAAAGATCCGGAAGAAATATCC**G**GGAC	
ADNg GABARAPL1 A549 KO GL1 c1 (All 2):		TATCGGAAAAAGGAAGGAGAAAAGATCCGGAAGAAATATCCGGACA	
ADNg GABARAPL1 A549 KO GL1 c3 (All 1):		TATCGGAAAAAGGAAGGAGAAAAGATCCGGAAGAAATATCCGGACA	
ADNg GABARAPL1 A549 KO GL1 c3 (All 2):		ATCGGAAAAAGGAAGGAGAAAAGATCCGGAAGAAATATCCGGACAG	

Sample	Start	Sequence	End
ADNg GABARAPL1 A549c :	336	GGGTCCCCGTGAGTGTAGAGGAGCGGAG	363
ADNg GABARAPL1 A549 KO GL1 c1 (All 1):		AGGGTCCCCGTGAGTGTAGAGGAGCGGAG	364
ADNg GABARAPL1 A549 KO GL1 c1 (All 2):		GGGTCCCCGTGAGTGTAGAGGAGCGGAG	
ADNg GABARAPL1 A549 KO GL1 c3 (All 1):		GGGTCCCCGTGAGTGTAGAGGAGCGGAG	
ADNg GABARAPL1 A549 KO GL1 c3 (All 2):		GGTCCCCGTGAGTGTAGAGGAGCGGAG	362

Sample	Start	Sequence	End
ADNg GABARAPL1 ACHN :	245	CGGTGCATCATGAAGTTCCAGTACAAGGAGGACCATCCCTTTGAG	289
ADNg GABARAPL1 ACHN KO GL1 cA (All 1):		CGGTGCATCATGAAGTTCCAGTACAAGGAGGACCATCCCTTTGA**A**	290
ADNg GABARAPL1 ACHN KO GL1 cA (All 2):		CGGTGCATCATGAAGTTCCAGTACAAGGAGGACCATCCCTTTG-G	288
ADNg GABARAPL1 ACHN KO GL1 cB (All 1):		CGGTGCATCATGAAGTTCCAGTACAAGGAGGACCATCCCTTTGA**A**	290
ADNg GABARAPL1 A549 KO GL1 cB (All 2):		CGGTGCATCATGAAGTTCCAGTACAAGGAGGACCATCCCTTTG-G	288

Sample	Start	Sequence	End
ADNg GABARAPL1 ACHN :	290	TATCGGAAAAAGGAAGGAGAAAAGATCCGGAAGAAATATCCGGACA	335
ADNg GABARAPL1 ACHN KO GL1 cA (All 1):	291	GTATCGGAAAAAGGAAGGAGAAAAGATCCGGAAGAAATATCCGGACA	336
ADNg GABARAPL1 ACHN KO GL1 cA (All 2):	289	TATCGGAAAAAGGAAGGAGAAAAGATCCGGAAGAAATATCCGGACAG	334
ADNg GABARAPL1 ACHN KO GL1 cB (All 1):	291	GTATCGGAAAAAGGAAGGAGAAAAGATCCGGAAGAAATATCCGGACA	336
ADNg GABARAPL1 A549 KO GL1 cB (All 2):	289	TATCGGAAAAAGGAAGGAGAAAAGATCCGGAAGAAATATCCGGACAG	334

Sample	Start	Sequence	End
ADNg GABARAPL1 ACHN :	336	GGGTCCCCGTGAGTGTAGAGGAGCGGAG	363
ADNg GABARAPL1 ACHN KO GL1 cA (All 1):	337	GGGTCCCCGTGAGTGTAGAGGAGCGGAG	364
ADNg GABARAPL1 ACHN KO GL1 cA (All 2):	335	GGTCCCCGTGAGTGTAGAGGAGCGGAG	362
ADNg GABARAPL1 ACHN KO GL1 cB (All 1):	337	GGGTCCCCGTGAGTGTAGAGGAGCGGAG	364
ADNg GABARAPL1 A549 KO GL1 cB (All 2):	335	GGTCCCCGTGAGTGTAGAGGAGCGGAG	362

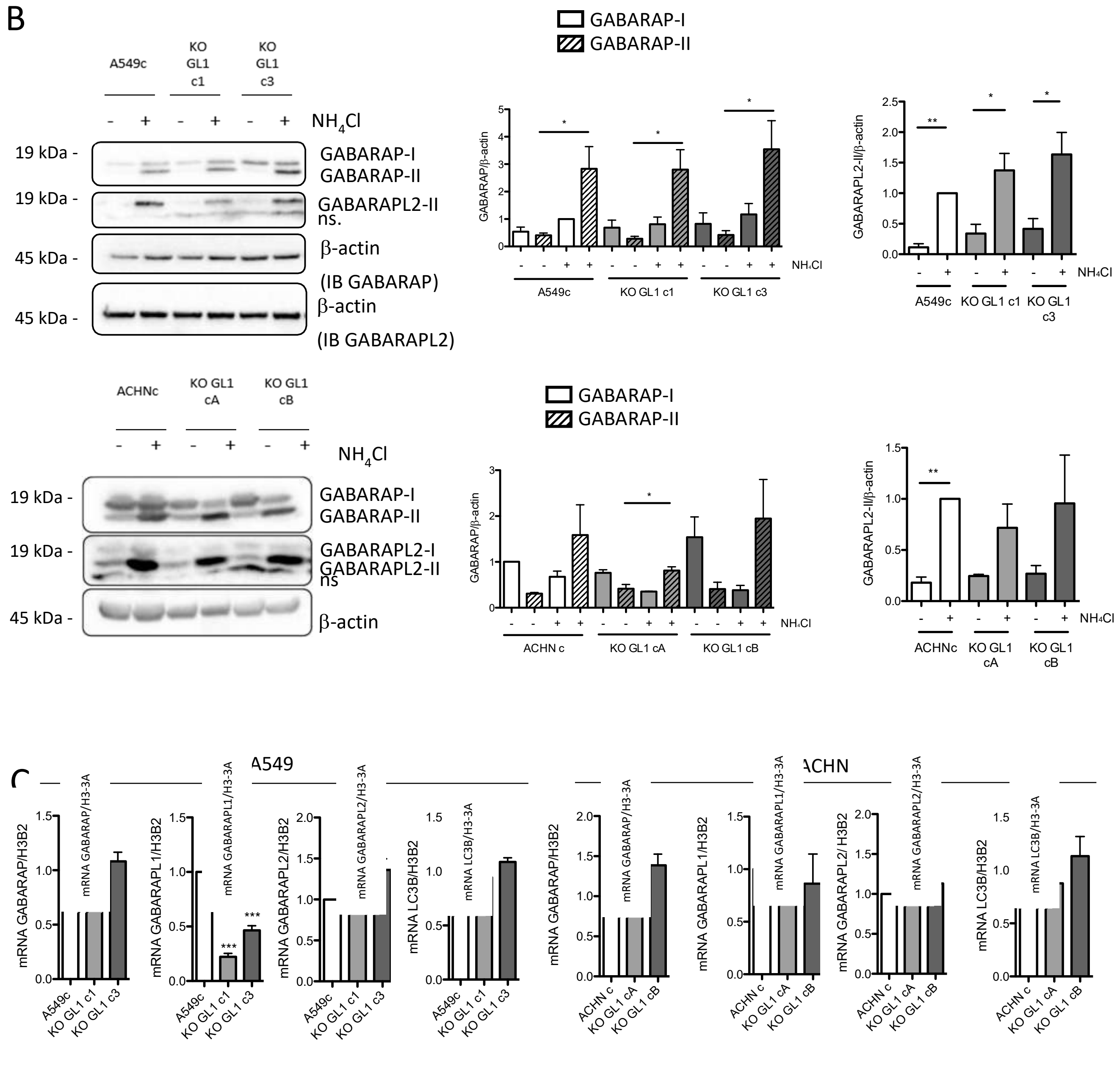

Figure S2.

(A) Sequencing analysis of A549c, A549 KO GL1 c1 and c3 using the following primers F-CGGTGCATCATGAAGTTCCA and R-CTCCGCTCCTCTACACTCAC for the A549c, KO GL1 c1, ACHN KO GL1 cA and KO GL1 cB; F-GCCCTGCGGTGCATCAT and R-CATCCCCTCCGCTCCTCTACACT for the A549c and KO GL1 c3. (B) Western blot analysis of GABARAP and GABARAPL2 levels in A549c, ACHNc and KO GABARAPL1 cells untreated or treated with 50 mM NH4Cl for 2 h. β-actin was used as a loading control. Protein levels were quantified using Image Lab. Data are represented as ±S.E.M. of at least three independent experiments. P*<0,05 compared to the control ; P-values were calculated using a Student's t test. (B) mRNA levels of GABARAP, GABARAPL1, GABARAPL2 and LC3B were measured by qRT-PCR in A549c, ACHNc and KO GABARAPL1 cells. The values calculated by the ΔΔCT method are relative to H3B2 levels and expressed as fold of change. Data are represented as ±S.E.M. of three independent experiments. P*<0,05 compared to the control ; P-values were calculated using a Student's t test.

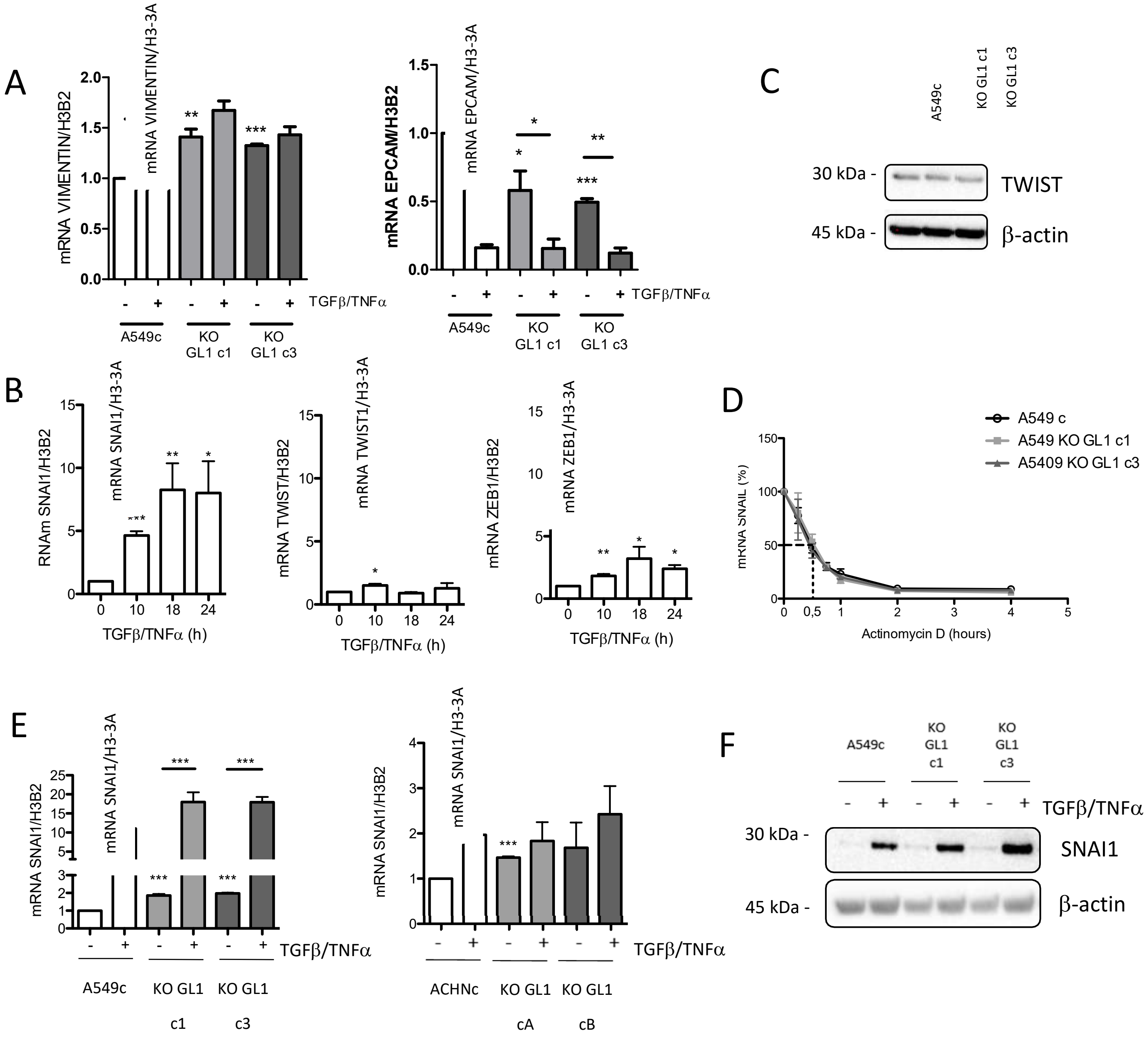
A
mRNA VIMENTIN/H3B2
mRNA VIMENTIN/H3-3A
TGFβ/TNFα
A549c
KO GL1 c1
KO GL1 c3
mRNA EPCAM/H3B2
mRNA EPCAM/H3-3A
C
A549c
KO GL1 c1
KO GL1 c3
30 kDa -
TWIST
45 kDa -
β-actin
B
RNAm SNAI1/H3B2
mRNA SNAI1/H3-3A
TGFβ/TNFα (h)
mRNA TWIST/H3B2
mRNA TWIST1/H3-3A
mRNA ZEB1/H3B2
mRNA ZEB1/H3-3A
D
A549 c
A549 KO GL1 c1
A5409 KO GL1 c3
mRNA SNAIL (%)
Actinomycin D (hours)
E
mRNA SNAI1/H3B2
mRNA SNAI1/H3-3A
A549c
KO GL1 c1
KO GL1 c3
ACHNc
KO GL1 cA
KO GL1 cB
F
A549c
KO GL1 c1
KO GL1 c3
TGFβ/TNFα
30 kDa -
SNAI1
45 kDa -
β-actin

Figure S3.

(A) mRNA levels of VIMENTIN and EPCAM were mesured by qRT-PCR in A549c cells untreated or treated with TGFβ (5 ng/ml) and TNFα (20 ng/ml) from 10 to 24 h. The value calculated by the ΔΔCT method are relative to H3B2 levels and expressed as fold of change. Data are represented as ±S.E.M. of three independent experiments. P*<0,05 compared to the control ; P-values were calculated using a Student's t test. (B) mRNA levels of SNAI1, TWIST, ZEB1, were measured by qRT-PCR in A549c cells untreated or treated with TGFβ (5 ng/ml) and TNFα (20 ng/ml) from 10 to 24 h. The value calculated by the ΔΔCT method are relative to H3B2 levels and expressed as fold of change. Data are represented as ±S.E.M. of three independent experiments. P*<0,05 compared to the control ; P-values were calculated using a Student's t test. (C) Western blotting analysis of TWIST level in A549c and KO GABARAPL1 untreated cells. β-actin was used as a loading control. Protein levels were quantified using Image Lab. Data are represented as ±S.E.M. of ten independent experiments. P*<0,05 compared to the control ; P-values were calculated using a Student's t test. (D) SNAI1 mRNA stability was studied by qRT-PCR in A549c and KO GABARAPL1 cells during actinomycin D (2,5 µg/ml) treatment for 0; 0,5; 0,75; 1; 2 and 4 h. The value calculated by the ΔΔCT method are relative to H3B2 levels and expressed as fold of change. Data are represented as ±S.E.M. of three independent experiments. P*<0,05 compared to the control ; P-values were calculated using a Student's t test. (E) mRNA levels of SNAI1 were measured by qRT-PCR in A549c, ACHNc and KO GABARAPL1 cells untreated or treated with TGFβ (5 ng/ml) and TNFα (20 ng/ml) during 24 h. The value calculated by the ΔΔCT method are relative to H3B2 levels and expressed as fold of change. Data are represented as ±S.E.M. of three independent experiments. P*<0,05 compared to the control ; P-values were calculated using a Student's t test. F. Western blotting analysis of SNAI1 level in A459c and KO GABARAPL1 cells untreated or treated with TGFβ (5 ng/ml) and TNFα (20 ng/ml) during 24 h. β-actin was used as a loading control. Protein levels were quantified using Image Lab. Data are represented as ±S.E.M. of three independent experiments. P*<0,05 compared to the control ; P-values were calculated using a Student's t test.

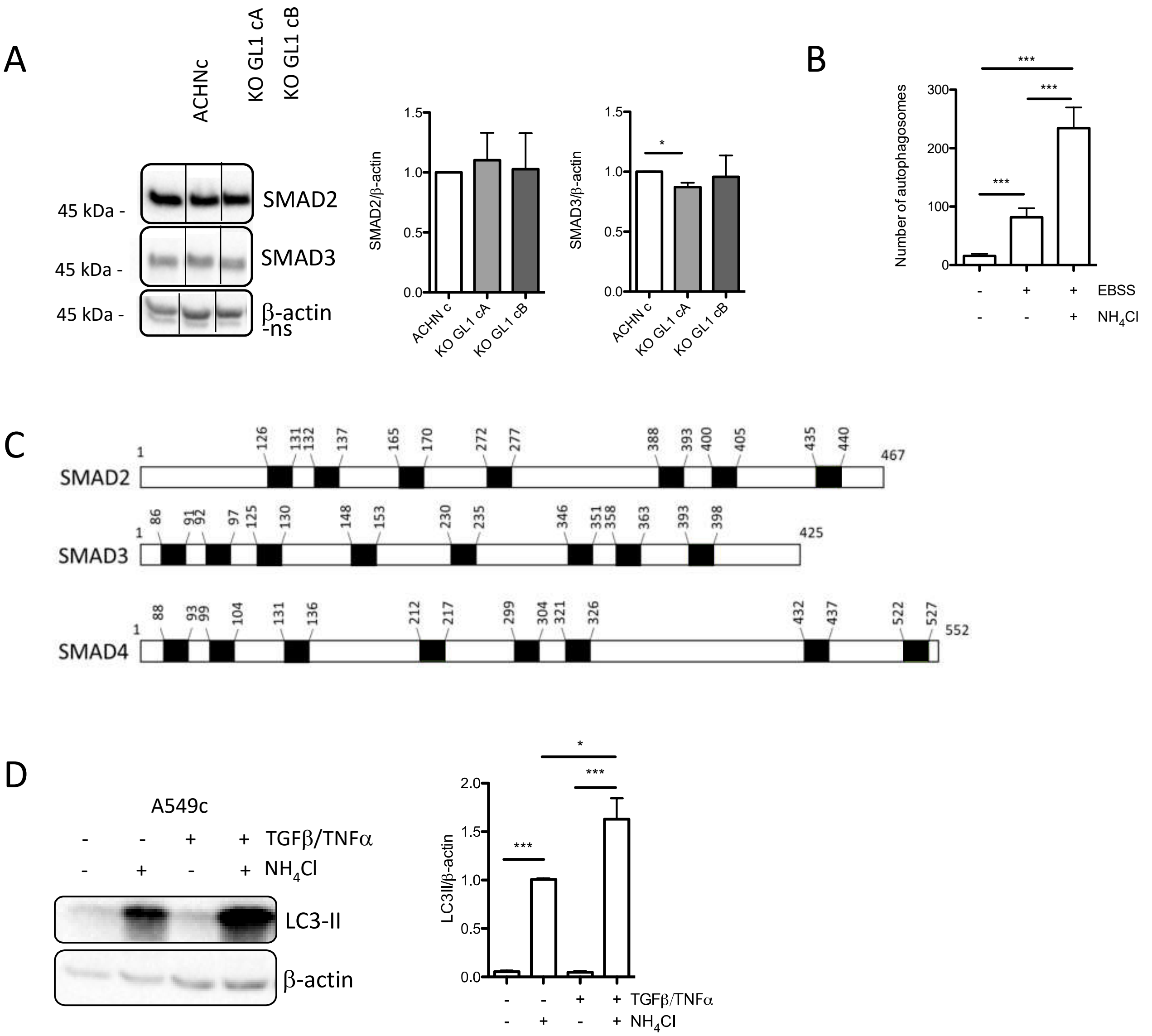

Figure S4.

(A) Western blotting analysis of SMAD2 and SMAD3 in ACHNc and ACHN KO GABARAPL1 cells. β-actin was used as a loading control. Protein levels were quantified using Image Lab. Data are represented as ±S.E.M. of three independent experiments. P*<0,05 compared to the control ; P-values were calculated using a Student's t test. (B) The number of autophagosomes in A549c cells transfected by pGFP-LC3 vector and cultured in complete medium or treated by EBSS for 4 h in presence or absence of 50 mM NH4Cl for 2 h has been quantify by confocal microscopy and analyzed using Image J software. Data are presented as a number of particles as ±S.E.M. of three independent experiments. P*<0,05 compared to the control ; P-values were calculated using a Student's t test. (C) Representation of potential AIM sequences in SMAD2, SMAD3 and SMAD4 proteins which have been identified using ILIR Autophagy Database. (D) Western blotting analysis of LC3B in A549c cells treated or not with TGFβ (5 ng/ml) and TNFα (20 ng/ml) during 24 h in presence or in absence of 50 mM NH4Cl. β-actin was used as a loading control. Protein levels were quantified using Image Lab. Data are represented as ±S.E.M. of three independent experiments. P*<0,05 compared to the control ; P-values were calculated using a Student's t test.

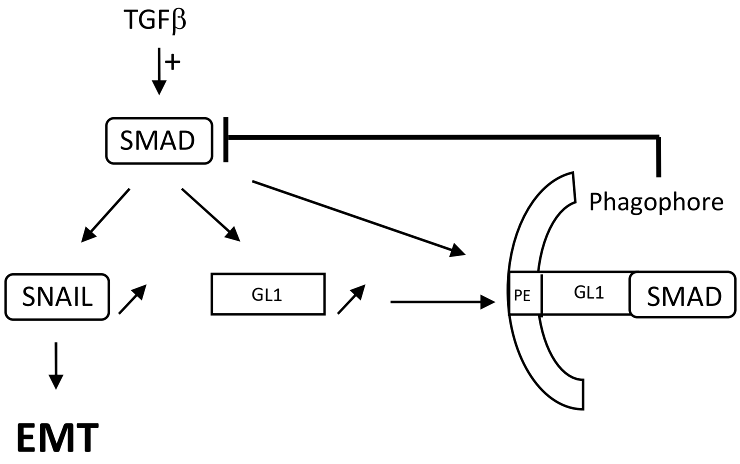

Figure S5.

Proposed model for GABARAPL1 negative feedback involvement in EMT regulation. First, TGFβ activated SMAD protein increasing SNAIL transcription inducing EMT. SMAD activation also conducts to the increase in GABARAPL1 protein implicates in the SMAD protein degradation inhibiting EMT.
